# Molecular diversity of genes related to biological rhythms (*period* and *timeless*) and insecticide resistance (*Na*_*V*_ and *ace-1*) in *Anopheles darlingi*

**DOI:** 10.1590/0074-02760220159

**Published:** 2023-07-10

**Authors:** Aline Cordeiro Loureiro, Alejandra Saori Araki, Rafaela Vieira Bruno, José Bento Pereira Lima, Simone Ladeia-Andrade, Liliana Santacoloma, Ademir Jesus Martins

**Affiliations:** 1 Fundação Oswaldo Cruz-Fiocruz, Instituto Oswaldo Cruz, Laboratório de Biologia, Controle e Vigilância de Insetos Vetores, Rio de Janeiro, RJ, Brasil; 2 Fundação Oswaldo Cruz-Fiocruz, Instituto Oswaldo Cruz, Laboratório de Biologia Molecular de Insetos, Rio de Janeiro, RJ, Brasil; 3 Fundação Oswaldo Cruz-Fiocruz, Instituto Oswaldo Cruz, Laboratório de Doenças Parasitárias, Rio de Janeiro, RJ, Brasil; 4 Instituto Nacional de Ciência e Tecnologia em Entomologia Molecular, Rio de Janeiro, RJ, Brasil; 5 Instituto Nacional de Saúde, Direção das Redes de Saúde Pública, Laboratório de Entomologia, Bogotá, Colômbia

**Keywords:** malaria vector, behavioural genes, insecticide resistance, population genetics

## Abstract

**BACKGROUND:**

Malaria is a public health concern in the Amazonian Region, where *Anopheles darlingi* is the main vector of *Plasmodium* spp. Several studies hypothesised the existence of cryptic species in *An. darlingi*, considering variations in behaviour, morphological and genetic aspects. Determining their overall genetic background for vector competence, insecticide resistance, and other elements is essential to better guide strategies for malaria control.

**OBJECTIVES:**

This study aimed to evaluate the molecular diversity in genes related to behaviour and insecticide resistance, estimating genetic differentiation in *An. darlingi* populations from Amazonian localities in Brazil and Pacific Colombian region.

**METHODS:**

We amplified, cloned and sequenced fragments of genes related to behaviour: *timeless* (*tim*) and *period* (*per*), and to insecticide resistance: voltage-gated sodium channel (*Na*_*V*_ ) and acetylcholinesterase (*ace*-*1*) from 516 *An. darlingi* DNA samples from Manaus, Unini River, Jaú River and Porto Velho - Brazil, and Chocó - Colombia. We discriminated single nucleotide polymorphisms (SNPs), determined haplotypes and evaluate the phylogenetic relationship among the populations.

**FINDINGS:**

The genes *per*, *tim* and *ace-1* were more polymorphic than *Na*_*V*_ . The classical *kdr* and *ace-1* ^*R*^ mutations were not observed. Phylogenetic analyses suggested a significant differentiation between *An. darlingi* populations from Brazil and Colombia, except for the *Na*_*V*_ gene. There was a geographic differentiation within Brazilian populations considering *per* and *ace-1*.

**CONCLUSIONS:**

Our results add genetic data to the discussion about polymorphisms at population levels in *An. darlingi*. The search for insecticide resistance-related mechanisms should be extended to more populations, especially from localities with a vector control failure scenario.

Malaria is one of the deadliest tropical diseases. In 2021, 247 million malaria cases occurred worldwide, and in the same year, Brazil recorded 163,585 cases.^1^ In this country, malaria prevails in the Amazonian Region, including the states of Acre, Amapá, Amazonas, Maranhão, Mato Grosso, Pará, Rondônia, Roraima and Tocantins.^2^ In Colombia, in 2020, 81,363 malaria cases were reported.^3^ Regarding the parasite, in Latin America, malaria is caused mainly by *Plasmodium vivax* and *Plasmodium falciparum* parasites, transmitted through infected *Anopheles* spp. females.^1^ *Anopheles (Nyssorhynchus) darlingi* Root 1926 is an important malaria vector in South America with a wide distribution from the south of Mexico to the north of Argentina and from the eastern side of the Andes Mountains to the Atlantic Coast.^4^ ^,^ ^5^ ^,^ ^6^ ^,^ ^7^ Specially in Colombia, *An. darlingi* is also found in the western side of the Andes.^8^

The primary tool for reducing the density of *Anopheles* spp. mosquito populations is based on neurotoxic insecticides, mainly indoor residual spraying (IRS) and long-lasting insecticide-treated nets (LLITN). Pyrethroids are the primarily used compounds globally.^1^ Brazilian and Colombian governmental campaigns deploy pyrethroids as the active ingredient in IRS-based applications and LLITN bednets in malaria-endemic regions. In addition, the organophosphate malathion also probably reaches *Anopheles* mosquitoes in urban centres because this compound has been used against arboviruses vectors in these regions.^9^ The intense and continuous use of chemicals selects insecticide-resistant mosquitoes, posing a severe threat to malaria control.^10^ ^,^ ^11^ There is a vast literature concerning insecticide resistance and the underlying molecular mechanisms in anophelines from African and Asian countries.^12^ ^-^ ^19^ On the other hand, the status of susceptibility or resistance to insecticides is scarcely known in *An. darlingi* populations, despite its importance in the cycle of malaria, especially in the Amazonian Region.

Pyrethroids and DDT target the voltage-gated sodium channel (*Na*_*V*_ ) of arthropods, causing the knockdown effect: “paralysis and consecutive death”.^20^ ^,^ ^21^ Single nucleotide substitutions in the *Na*_*V*_ gene related to resistance to the knockdown effect are referred to as *kdr* mutations.^7^ ^,^ ^22^ ^,^ ^23^ The substitutions Leu to Phe or Ser in the codon 1014 are the most found *kdr* mutations in diverse *Anopheles* species,^24^ including the neotropical *An. albimanus* and *An. albitarsis* s.s.^18^ ^,^ ^25^ Organophosphates (OPs) and carbamates target the acetylcholinesterase enzyme, whereas mutations in the *ace-1* coding gene are associated with resistance to OPs insecticide class. The substitution G119S in the *ace-1* gene was found in resistant *Culex pipiens* ^26^ ^,^ ^27^ and species of the *Anopheles gambiae* complex cryptic species.^26^ ^,^ ^28^ Analyses of the nucleotide diversity spanning these mutations are essential to tracking the evolutionary dynamics of arising and dispersal of insecticide resistance mechanisms.^13^ ^,^ ^16^ ^,^ ^29^ ^,^ ^30^ ^,^ ^31^

The broad geographic occurrence of *An. darlingi*, associated with differentiation in behavioural, morphological, and genetic traits,^4^ ^,^ ^32^ ^,^ ^33^ raised the hypothesis that this species would comprise a group of cryptic species. For instance, there are reports describing variations in the egg (exochorion) morphology,^34^ wings morphometry,^35^ ^,^ ^36^ ^,^ ^37^ hematophagy activity behaviour, and lifespan.^38^ ^,^ ^39^ Besides, polymorphism in the banding patterns of polytene chromosomes also suggested cytogenetic variations.^40^ ^,^ ^41^ ^,^ ^42^ At a molecular level, several markers showed diversity among populations along with geographic variation, such as *ITS2* in rDNA,^43^ *cytochrome oxidase I* gene (COI) in mtDNA,^44^ ^,^ ^45^ microsatellites^46^ ^-^ ^51^ and single nucleotide polymorphisms (SNPs).^4^ ^,^ ^52^ ^,^ ^53^ Despite these multiple behavioural, morphological, and genetic distinctions, some studies claim that these differences are divergences among populations, not enough to evidence *An. darlingi* as a complex of cryptic species.^4^ ^,^ ^35^ ^,^ ^36^ ^,^ ^38^ ^,^ ^45^ ^,^ ^47^ ^,^ ^50^ ^,^ ^51^ ^,^ ^54^ ^-^ ^56^

Genes that influence biological rhythm activities, like mating and locomotion,^57^ ^,^ ^58^ are helpful molecular markers to evidence insect speciation. In this context, the genes *timeless* (*tim*) and *period* (*per*) are good examples in population genetic studies with *Drosophila* and also insects of sanitary importance, including *Anopheles*.^59^ ^,^ ^60^ Analysis of nucleotide variation in *tim* supported the division of *Anopheles (kertesia) cruzii* into two cryptic species, where one group occurs in Bahia State (northeast of Brazil) and the other in the Southern and Southeastern Brazilian regions.^61^ In the *Nyssorhynchus* subgenus, *An. triannulatus* s.l. was divided into two distinct clusters: one comprising *An. halophylus* and *An. triannulatus* species C, and the second represented by *An. triannulatus* s.s.^62^ Variations in the *per* gene, summed with a series of biochemical and behavioural observations, contributed to separating the species *Lutzomyia longipalpis*, the primary sandfly vector of *Leishmania infantum* in Brazil, into several groups related to courtship song.^59^ ^,^ ^63^ Populational studies with other sandflies, *Lutzomyia umbratilis*, *Lutzomyia intermedia*, and *Lutzomya whitmani* corroborated the *per* gene as an excellent molecular marker for the analysis of speciation processes.^64^ ^,^ ^65^ ^,^ ^66^

Discrimination among cryptic species of insect vectors has great epidemiological importance once different species may have distinct vector capacity, therefore requiring specific vector control strategies.^67^ This study explored the molecular diversity of genes related to behaviour and insecticide resistance, estimating genetic differentiation in *An. darlingi* populations from Brazilian and Colombian localities.

## MATERIALS AND METHODS

**Fig. 1:**
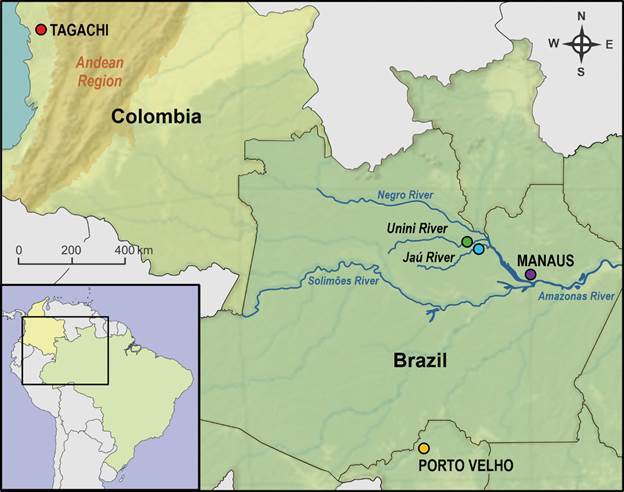
*Anopheles darlingi* sample sites. In red: Tagachi-Quibdó, Choco Department - Colombia. In green, blue, and purple: Unini River, Jaú River, and Manaus, respectively, in Amazonas State. In yellow: Porto Velho, Rondônia State. The Colombian Andean Region is indicated.

*Samples description* - We obtained female *An. darlingi* samples from five Amazonian localities: Estrada do Brasileirinho, Manaus (79 samples), Amazonas State (3º01’16’’S, 59º52’55’’W); Unini River (125 samples) (01º45’46.0’’S, 62º13’39.6’’W) and Jaú River (132 samples) (01º53’2.0’’S, 61º44’31,6’’W), Barcelos, Amazonas State; and Porto Velho (125 samples), Rondônia State (7º18’32’’S, 67º05’42’’W) in Brazil; and from Tagachi-Quibdó (55 samples), Chocó Department (6º7’53.04’’N, 76º26’0.6’’W) in Colombia (Fig. 1). These samples were collected with a Castro aspirator by gently capturing females seeking blooding feeding in protected human landing catches (HLC) during the first hours of the evening.^68^ Mosquitoes were identified based on morphological characters.^69^ Those confirmed as *An. darlingi* were preserved in silica or ethanol and shipped to the Laboratory for further genomic analyses.

We calculated geographic distances among the localities using the free software Qgis, version 2.18.24 (available from: http://www.qgis.org/). We highlight that the collections were not designed specifically for this study, and that the samples were kindly provided for the molecular assays and analyses herein presented. In general, captures occurred indoors, around houses, and in adjacent forest areas.

*DNA extraction and sample pooling* - Genomic DNA was individually extracted from adult females following^70^ with slight modifications. Mosquitoes were individually macerated in 200 µL TNES buffer [250 mM Tris pH 7.5, 2 M NaCl, 100 mM EDTA, and 2.5% sodium dodecyl sulphate (SDS)] with the addition of 2 µL of 20 mg/mL proteinase K and left for incubation in a 56ºC water bath overnight. After 1 minute centrifugation, we added 100 µL of 5 M NaCl to precipitate the protein content and centrifuged again for 6 min at 15,000 g. The supernatant was transferred to new tubes with a similar volume of 100% isopropanol and centrifuged for 5 min at 15,000 g. The supernatant was discarded, and the DNA pellet was washed with 70% ethanol by 6 min centrifugation at 15,000 g and supernatant discarding. To remove any alcoholic trace, we heated the open tubes for 10 min at 60ºC. Finally, DNA was eluted in TE 0.1X (30 µL) and quantified in a NanoDrop One (ThermoFisher). We made a DNA pool for each population (Manaus, Unini River, Jaú River, Porto Velho and Colombia): 1 µL (around 0,07 µg) of each DNA sample of this respective population: Manaus (N = 79), Unini River (N = 125), Jaú River (N = 132), Porto Velho (N = 125), and Colombia (N = 55). Samples originated in Tagachi - Chocó are referred as Colombia in this paper.

*Polymerase chain reaction (PCR) amplification* - The primers for *Na*_*V*_ , *ace*-1, and *per* fragments were designed for this study, whereas the *tim* fragment primers were previously available [Supplementary data (Table I)]. PCR amplifications were carried out with one of the three hi-fidelity polymerase kits options: 1) Phusion High-Fidelity PCR Master Mix with GC Buffer (New England, BioLabs); 2) USB Fidelitaq^TM^ DNA Polymerase (Affymetrix); or 3) Pfu DNA Polymerase (Thermo Scientific), following manufacture instructions and annealing temperature as described in Supplementary data (Table I). All reactions were run in a Veriti Thermocycler (Applied Biosystems), and the PCR products were purified using the magnetic beads kit Agencourt AMPure XP (Beckman Coulter), according to the manufacture instructions. We cloned the purified products with the Kit pJet (Fermentas) into *Escherichia coli* DH5-α competent cells. The DNA preparations followed the alkaline lysis procedure,^71^ and sequencing reactions were performed using the *kit* BigDye Terminator v3.1 Cycle Sequencing (Applied Biosystems) following standard procedures and submitted to an ABI Prism 3730 (Applied Biosystems) in the DNA Sequencing Facility Platform at Fiocruz.

*Sequence analyses* - We used the software Geneious 9.1.8^72^ to edit, identify, align, annotate and translate the obtained sequences. The term haplotype is here used to refer to the distinct sequences of the same gene fragment. The polymorphism analyses identified the number of haplotypes (h), polymorphic sites (S), nucleotide diversity (Π), neutral parameter (Ө). We carried out three tests of selective neutrality: Tajima’s D (Tajima 1989), Fu’s Fs (Fu 1997) and Ramos-Onsins and Rozas’ R 2 (2002) with the software DnaSP 5.0.^73^ For the genetic differentiation analyses, we used the software ProSeq 2.9.1^74^ for calculating fixation index (*Fst)*, number of polymorphic sites (Ss), fixed sites (Sf), and exclusive polymorphic site (Sx, Sy). A Mantel test on geographical [estimated as Ln (km)] and genetic distance [estimated as *Fst*/(1-*Fst*)] was performed in Arlequin 3.1.^75^ The correlation coefficient (r) was estimated using 10,000 permutations. To construct phylogenetic relationships and choose the best nucleotide substitution model, we used the JModel Test 2.0.^76^ The maximum likelihood (ML) trees were obtained with MEGA 7.0,^77^ because of its better resolution, and the haplotype networks were achieved using TCS analysis with the software PopART.^78^ Bayesian inferences were carried out using MrBayes 3.2.4.^79^ With this software, we performed for 10-million generation with two parallel searches using nine heated and one cold Markov chain (MCMC). The visualisation and analysis of the MCMC trace files generated through Bayesian phylogenetic inference were performed using Tracer v.1.7.2.^80^ Convergence of the two runs (average standard deviation of split frequencies < 0.01) and likelihood stationarity were checked. Trees were exported to FigTree v1.4.3^81^ for visualisation.

## RESULTS

We evaluated DNA sequences of fragments of the genes *per*, *tim*, *Na*_*V*_ and *ace-1*, obtained from a total of 516 specimens of *An. darlingi* from Amazonian localities in Brazil and Colombia. The haplotypes unrevealed of each gene are accessible in the Genbank [sequences and respective accession numbers in the Supplementary data (Table II) (*per*), Table IV (*tim*), Table V (*Na*_*V*_ ) and Table VI (*ace-1*)].

Polymorphism analyses

*Biological rhythm genes* - We obtained 101 sequences with a 535 bp fragment of the gene *per*. The fragment included two exons (exon 2 = 22 bp and exon 3 = 437 bp) and an intron (76 bp). We observed 39 variable sites (7%), resulting in 65 haplotypes [Supplementary data (Table II)] with 26 synonymous substitutions and 13 non-synonymous: two and 11, respectively, in exons 2 and 3. Amino acid changes [Supplementary data (Fig. 1)] occurred at the alignment positions: 3 (Leu/Ser), 4 (Asp/Tyr), 15 (Thr/Lys), 18 (Thr/Met), 39 (Gly/Glu), 57 (Gly/Asp), 89 (Gly/Glu), 95 (Arg/Cys), 97 (Val/Ala), 107 (Ser/Leu), 108 (Ser/Asn), 141 (His/Gln), 144 (His/Asn). Supplementary data (Table III) summarises the nucleotide variation for each population, evaluations of the number of haplotypes (H, from 11 to 20), number of polymorphic sites (S, 18 - 23), nucleotide diversity (Π, 0.007 up to 0.011), and neutral parameter (Ө, 0.009 - 0.012). The diversity index was similar in the five populations; nonetheless, the diversity of haplotypes (0.852 up to 0.995), considering the number of sequences at each locality, was higher in Porto Velho (95.2%) and Manaus (89.5%) and lower in Unini River (70.6%), Colombia (56.5%) and Jaú River (52.4%). Most of the haplotypes (89%) were exclusive to one population: Unini River (n = 7), Jaú River (n = 8), Manaus (n = 13), Porto Velho (n = 17) and Colombia (n = 13). Considering all sequences, the *per-4* haplotype [Supplementary data (Table II)] was the most frequent, represented by nine sequences: eight in Jaú River and one in Unini River, followed by *per-1* with eight sequences: six in Unini River, one in Manaus, and one in Porto Velho.

**Fig. 2:**
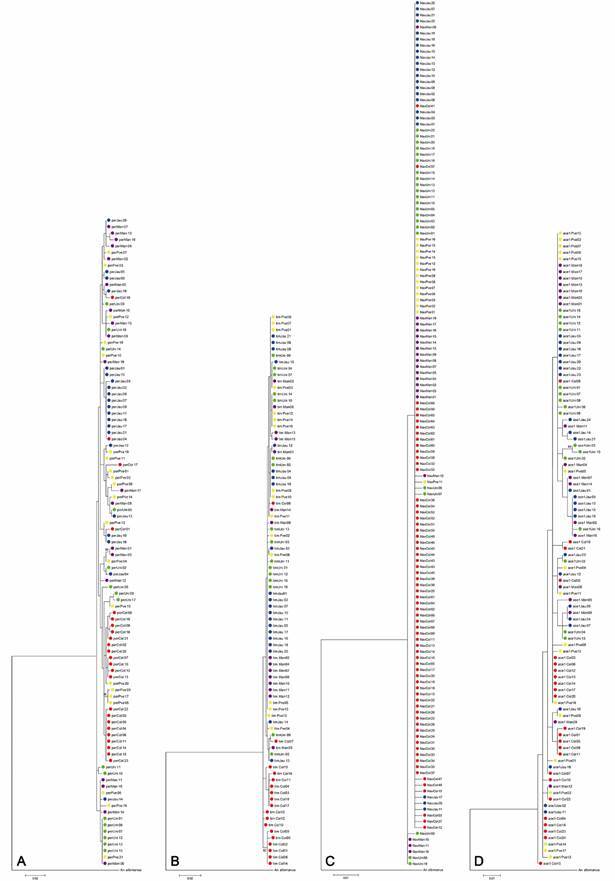
phylogenetic trees to *per* (A), *tim* (B), *Na*_*V*_ (C) and *ace-1* (D). Numbers on nodes represent the percentage bootstrap values on 1000 replications. In green: Unini River (Uni), blue: Jaú River (Jau), yellow: Porto Velho (Pve), purple: Manaus (Man); red: Colombia (Col).

The 737 bp *tim* sequenced fragment included two exons (177 and 142 bp, respectively, exons 3 and 4) and two introns (82 and 336 bp, respectively, introns 3 and 4). In the total of 86 sequences, we observed 28 polymorphic sites (~ 3,8%), resulting in 34 haplotypes [Supplementary data (Table IV)], with three non-synonymous substitutions, all in exon 3. Amino acid changes [Supplementary data (Fig. 2)] occurred at three different alignment sites: 10 (Glu/Arg), 41 (Gly/Asp), and 55 (Thr/Ala), all in Colombia. Supplementary data (Table III) outlines nucleotide variation for each population (H: 8-12, S: 8-18, Π: 0.002-0.006 and Ө: 0.003 - 0.007). As observed in *per*, Brazilian populations showed a similar number of polymorphic sites and haplotype amount in the *tim* sequences, less diverse than in the Colombian population. The diversity indexes were similar in all populations, and the diversity of haplotypes was higher in Colombia (66.7%), followed by Unini River and Porto Velho (62.5%), Manaus (60%), and Jaú River (38.1%). Most of the haplotypes (76.5%) were exclusively found in one population: Colombia (12), Manaus (4), Unini River (4), Jaú River (3), and Porto Velho (3). The haplotype *tim-1* was the most frequent (23 sequences): ten in Jaú River, seven in Manaus, four in Unini River, and two in Porto Velho, followed by *Tim-6* (7 sequences): three in Jaú River and Porto Velho and one in Unini River [Supplementary data (Table IV)].

*Insecticide resistance genes* - The *Na*_*V*_ fragment included two exons (88 and 177 bp, respectively, in exons 20 and 21) and one intron (74 bp). In the 143 obtained sequences, we observed 16 polymorphic sites (~ 13%), resulting in 17 haplotypes [Supplementary data (Table V)]. Amino acid changes [Supplementary data (Fig. 3)] occurred at ten different alignment sites: 5 (Trp/Arg), 10 (Val/Ala), 16 (Ile/Thr), 17 (Pro/Leu), 40 (Ser/Pro), 53 (Asp/Asn), 66 (Ile/Thr), 68 (Arg/Cys), 69 (Phe/Leu), 79 (Asn/Asp). When numbered according to the *Musca domestica* sodium channel protein, these substitutions are usually W991R, V996A, I1002T, P1003L, S1026P, D1039N, I1052T, R1054C, F1055L, N1065D, respectively, none of which ever related to some known *kdr* mutation. Supplementary data (Table III) summarises nucleotide variation for each population (S: 14-66, H: 2-7, Π: 0.000-0.001, Ө*:* 0.000 - 0.003). In general, nucleotide variation was low and similar amongst populations. Unini River presented the highest diversity of haplotypes (22.7%), followed by Manaus (21.1%), Jaú River (18.2%), Porto Velho (14.3%), and Colombia (10.6%). Most haplotypes were exclusively found in one population (88.2%): Colombia (6), Unini River (3), Jaú River (3), Manaus (2), and Porto Velho (1). The haplotype *Na*_*V*_ *-1* was the most frequent, with 122 sequences: 60 in Colombia (49.2%), 19 in Jaú River (15.5%), 17 in Unini River (13.9%), and 13 in Manaus and Porto Velho (10.7%). The second most frequent was *Na*_*V*_ *-9*, with five sequences: three in Manaus (60%) and two in Jaú River (40%) [Supplementary data (Table V)].

The 344 bp sequenced fragment of *ace-1* included one exon (exon 5), represented by 99 sequences with 25 polymorphic sites (~ 7%), resulting in 36 haplotypes [Supplementary data (Table VI)]. Amino acid changes occurred at four different alignment sites [Supplementary data (Fig. 4)]: 2 (Val/Ile), 28 (Met/Thr), 55 (Ala/Ser), and 107 (Glu/Val). According to the amino acid numbering in the acetylcholinesterase protein of *M. domestica*, these substitutions are V57I M83T, A110S, and E162V, respectively, none so far reported related to organophosphates and carbamates resistance. Supplementary data (Table III) outlines nucleotide variation for each population (S: 8-11, H: 8-13, Π: 0.006-0.007, Ө, 0.006-0.009). Porto Velho presented the highest haplotype diversity (70.6%), followed by Manaus (55.6%), Jaú River (54.2%), Unini River, and Colombia (50%). Most of the haplotypes (63.9%) were exclusively found in one population: Colombia (6), Unini River (6), Porto Velho (5), Jaú River (4), and Manaus (3). The haplotype *Ace-5* was the most frequent [Supplementary data (Table VI)], represented by 28 sequences: eight in Unini River (28.5%), seven in Jaú River and Manaus (25%), five in Porto Velho (18%), and one in Colombia (3,5%). *Ace-2* was the second most frequent haplotype, with eight sequences: two in both Jaú River and Porto Velho and four in Colombia. None of these haplotypes carried the *ace-1* mutant allele (119S), providing no evidence of *ace-1* duplication.

*Selective neutrality test* - The neutrality tests indicated that in all cases, values were not significant after Bonferroni’s correction, indicating no obvious departures from neutrality [Supplementary data (Table VII)].

Genetic differentiation

*Biological rhythms genes* - Analyses with the gene *per* detected significant *F*_st_ values (p < 0.001), higher than 0.15 in all comparisons between Colombia or Jaú River with all other populations [Supplementary data (Table VIII)]. Interestingly, populations from Manaus and Porto Velho, which are around 940 km apart, showed a low *F*_s_t (0.027; p > 0.05), while Unini River and Jaú River populations, distant in around 5 km, presented a significant differentiation (*F*_st_ = 0.190, p < 0.001). In general, Colombian was the most differentiated population (*F*_st_ = 0.208 - 0.475; p < 0.001), followed by Jaú River (F_st_ = 0.178 - 0.190; p < 0.001) [Supplementary data (Table VIII)]. There were no fixed substitution sites in all comparisons [Supplementary data (Table IX)]. We observed between two and 13 exclusive variable polymorphisms, mostly in the Colombian population. In short, the *per* fragment differed Colombian from Brazilian populations and separated Jaú River from the other Brazilian ones.

Regarding the *tim gene*, all comparisons involving *An. darlingi* populations from Brazil showed low genetic differentiation. However, all populations compared with the Colombian presented significant *F*_st_ values (p < 0.001), ranging from 0.347 to 0.389 [Supplementary data (Table VIII)]. No fixed substitution sites were found. Like the *per* gene, more exclusive polymorphic sites were observed in the comparisons between the Colombian and Brazilian populations. Hence, *tim* gene analyses also differ *An. darlingi* populations from Colombia and Brazilian.

*Insecticide resistance genes* - All *Na*_*V*_ gene fragment comparisons did not show any significant genetic differentiation between Brazilian pairwise populations (*F*_st_ from 0 to 0.06; p > 0.05). The Colombian population differed with Unini River (*F*_st_ = 0.035, p < 0.01) and with Manaus (*F*_st_ = 0.057, p < 0.05) populations [Supplementary data (Table VIII)]. There was one shared polymorphic site between Unini River and Manaus (Supp. Material Table IX), no one fixed substitution site and low exclusive variation in all populations.

In general, the *ace-1* gene evidenced low and non-significant *F*st values, suggesting low genetic differentiation among Brazilian populations. The exception was the pair Unini River and Porto Velho populations, that although they are nearly 1,130 km apart, showed a significant differentiation (*F*_st_ = 0.164, p < 0.001) [Supplementary data (Table VIII)]. All comparisons between the Colombia and a Brazilian populations resulted in high and significant differentiation index values (*F*_st_ = 0.054 - 0.330, p < 0.05). There were elevated shared polymorphic sites, no fixed substitution sites, and low exclusive variable sites among all populations [Supplementary data (Table IX)]. Like with the biological rhythms genes, *ace-1* analyses differentiated the *An. darlingi* from Colombia from all Brazilian populations.

*Geographic x genetic differentiation* - The Mantel test [Supplementary data (Tables X-XI)] did not evidence any statistically significant correlation between genetic and geographical distances among Brazilian populations, therefore not supporting the hypothesys of Isolation by Distance model. Nevertheless, we observed positive correlations for *tim* (p = 0.024) and with a p near to significance value in *ace* (p = 0.057) when Colombia was included.

*Phylogenetic analysis* - The TCS haplotype networks in most cases separated the Colombian from the other populations, except for *ace-1* [Supplementary data (Figs 5-8)] . We carried out ML analysis and Bayesian inference of phylogenetic trees. The ML (Fig. 2) and Bayesian phylogenetic trees [Supplementary data (Figs 9-12) considered the JModel Test, with the best-fit models of nucleotide substitution: General Time Reversible model for *per* fragment, Kimura 2 parameters for *tim*, Hasegawa-Kishino-Yano model for *Na*_*V*_ gene and Tamura 3 parameters for *ace*-1. The Bayesian phylogenetic trees of *per* and *tim* genes showed a clustering of most Colombian sequences with posterior probabilities of 0.8083 and 0.9967, respectively [Supplementary data (Figs 9, 10)]. No sub-clustering among Brazilian populations was observed. For the insecticide resistance-related genes, most *Na*_*V*_ sequences were grouped homogeneously, showing a low variation level as expected, once it is known that the *Na*_*V*_ is highly conserved, even among distinct taxa. However, *ace-1* showed heterogeneous distribution with no supported clusters.

## DISCUSSION

The hypothesis that *An. darlingi* is a complex of cryptic species still needs more robust support, despite several studies evidencing behavioural, morphological, biochemical and genetic differences.^4^ ^,^ ^6^ ^,^ ^35^ ^,^ ^36^ ^,^ ^38^ ^,^ ^44^ ^,^ ^47^ ^,^ ^50^ ^-^ ^52^ ^,^ ^55^ ^,^ ^56^ In this study, we presented evidence of genetic differentiation among *An. darlingi* Amazonian populations from Brazil and a population of Tagachí in the department of Chocó - Colombia, with genes commonly used in molecular behaviour studies and markers for insecticide resistance mechanisms.

As a whole, the genes *per*, *tim* and *ace-1* showed similar polymorphic diversity, with low nucleotide diversity indexes (0.00257-0.01171). The gene *per* is also related to sexual selection^82^ and is expected to be highly conserved among distinct populations. Indeed, populational studies with other insect disease vectors also showed low nucleotide diversity in the *per* gene, as in *An. gambiae* s.l. populations from Burkina Faso, Senegal and Cameroon (Π 0.0031)^82^ and the sandfly *Lutzomya umbralitis* (Π 0.0031) from Manacapuru, in Amazonas State.^66^ The di-aminoacid repetition Thr-Gly in the Period protein of *D. melanogaster* is related to thermostability, and the frequency of haplotypes with a specific number of repetitions varies according to geographic origin in the Northern hemisphere (Thr-Gly_17_ and Thr-Gly_20_). The Thr-Gly_20_ repetition block was suggested to be an adaptation to cold weather and high latitudes, while Thr-Gly_17_ is adapted to hot weather, as they are more frequent in the Mediterranean Region.^83^ ^,^ ^84^ ^,^ ^85^ Our study detected a block of repetitions with the CAT codon in the *An. darlingi per* gene, coding for 12 histidines (His_12_) in all *per* haplotypes. However, there was a substitution inside this poly-His region in the haplotypes *per34* from Colombia (His to Glu) and *per63* from Manaus (His to Asn). It will be interesting to further elucidate the phenotypical meaning of this polymorphism.

Polymorphisms found in the *tim* gene contributed to identifying sibling cryptic species in sympatric samples of the Triannulatus complex, divided into *An. triannulatus* s.s., *An. halophylus* and *An. triannulatus*, in the central part of Brazil.^62^ Herein we showed similar diversity indexes for *tim* in all evaluated populations, however with high and significative *F*_st_ pairwise values differentiating Brazilian and Colombian populations. The *tim* polymorphism analyses in the main simian and human *Plasmodium* vectors found in the Atlantic Rain Forest in the Brazilian littoral evidenced the division of *An. (Kerteszia) cruzii* into one distinct cluster in each Northeastern and Southeastern Regions.^61^ To our knowledge, the only previous analyses of this gene in *An. darlingi* explored genetic differences in the host seek biting behaviour of endophily and exophily in Portuchuelo and Macapá, in the northeastern Amazon, with low but relevant differences between these two groups.^86^ Further studies focusing on the variation of *tim* in more *An. darlingi* populations, considering geologic and climate aspects, will help to elucidate those findings.

The *Na*_*V*_ fragment revealed the lowest nucleotide diversity (Π, 0.00042 - 0.00173) compared to the other genes. A previous study on this gene showed low nucleotide variability in *Lu. longipalpis* and *Lutzomyia cruzi*, with specific variations in each species.^87^ It is expected because few mutations are permissive in the *Na*_*V*_ protein, given this highly conserved functional role in the physiology of the nervous system.^88^ Unlike *per* and *tim*, *Na*_*V*_ and *ace-1* presented some similar haplotypes between Brazilian and Colombian mosquitoes suggesting gene flow among them and ancestral polymorphism. Interestingly, the Porto Velho population revealed two highly polymorphic *Na*_*V*_ haplotypes, with all variation observed in the intron. Future studies in that locality, considering collections under distinct circumstances (inside/outside the houses, different moments along the night, etc.), and including samples from other sites will provide better clues whether this polymorphism is linked to different behavioural aspects.

Neurotoxic insecticide usage is the primary strategy in vector control, mainly because of the use of LLITN, which reduced significantly malaria cases in Africa.^89^ Resistance management studies associated with vector population genetics are essential to understand better the origins and dispersion of resistance mechanisms.^67^ The low nucleotide diversity profile in the *ace-1* gene that we observed in *An. darlingi* populations match similar profiles observed among populations of *An. gambiae* s.l. (Π 0.00634)^90^ and *Culex pipiens* (Π 0.024).^91^ Gene duplication events on *ace-1* were described in *Anopheles* and *Culex* species.^26^ ^,^ ^90^ ^,^ ^91^ ^,^ ^92^ This phenomenon may enhance genetic diversity in this gene, as the different copies may evolve independently. The *ace-1* ^*R*^ allele (G119S), associated with organophosphate resistance, also results in a high fitness cost to insect development and reproduction.^90^ Continued pressure has selected duplications containing the wild-type allele and an “organophosphate-resistant” allele, thus maintaining insecticide resistance and decreasing deleterious fitness effects.^92^ This same organophosphate resistance has not been described in Amazonian populations of *An. darlingi*,^93^ however the *ace-1* ^*R*^ allele was found in *An. albimanus* from Peru.^93^ Although this mutant allele was not found among our samples, the high diversity observed and the presence of at least non-synonymous substitutions suggest that we should monitor variations in the insecticide susceptibility in these populations, especially in those closer to urban centres where organophosphates are used against *Aedes* mosquitoes.^9^

The classic L1014F *kdr* mutation is reported in at least 14 *Anopheles* species, including the Neotropical *An. albitarsis* s.s. in southern Brazil^25^ and on *An. albimanus* in Central and North America,^18^ ^,^ ^94^ selected due to the intense pressure exerted by vector control interventions with pyrethroids in IRS and LLITN^24^ or in proximity to agricultural areas.^25^ We did not find any non-synonymous variation in the *Na*_*V*_ gene fragment of *An. darlingi*. Further studies must consider evaluating the sequence beyond the fragment containing the 1014 site in the *Na*_*V*_ genes, as the substitution Asn to Tyr found in the 1575 *Na*_*V*_ site of *An*. *gambiae*, for example.^16^ ^,^ ^24^ Due to the *kdr* recessive trait, the mutant allele tends to remain under low frequencies for a long time, until it reaches the *tipping-point*, from which the *kdr* frequency will increase under exponential speed.^95^ The surveillance of known *kdr* mutations and screenings in search for non-described variations in the *Na*_*V*_ gene are essential to be investigated in *An. darlingi* populations before *kdr* alleles reach levels able to affect the chemical control efficacy. We must also consider that in several regions, *An. darlingi* populations are exophilic and exophagic,^6^ ^,^ ^96^ ^,^ ^97^ ^,^ ^98^ meaning that the mosquito contact with insecticide in IRS and impregnated nets can be minimal. Therefore, future studies should consider testing samples collected indoors and outside the houses. Additionally, recent studies have shown differences between *An. darlingi* populations from urban and rural areas due to human interventions, deforestation and seasonal climate variations influencing on mosquito distribution. These changes may impact directly on vector control and surveillance strategies.^54^ ^,^ ^97^

A previous study with *An. darlingi* populations throughout Central and South American localities using sequences of COI fragment evidenced a well distinguished differentiation of the pool of this mitochondrial DNA haplotypes from Latin American and South American populations.^44^ Here, analyses with *per*, *tim* and *ace-1* fragments grouped separated Brazilian and Colombian *An. darlingi* populations. The Mantel Test evidenced significant isolation by distance, at least for the *tim* and *ace-1* fragments among Brazilian and the Colombian populations. Our samples from Colombia are from a locality in the western Andean Region (Fig. 1), and a previous study demonstrated that the Andes mountain is a potent barrier that prevents the geneflow between *An. darlingi* populations from eastern and western sides of these montains.^56^ The lack of gene flow and distinct selection processes through time is an example of a pathway toward speciation, as discussed in previous studies.^4^ ^,^ ^37^ ^,^ ^44^ ^,^ ^48^ ^,^ ^50^ ^,^ ^51^ For instance, wing morphological variations observed in *An. darlingi* between indoor and outdoor mosquitoes in Colombia’s western and eastern regions were attributed to incipient species differentiation.^37^ The Amazonian hydrographic basin is also an important barrier to the geneflow, as demonstrated by microsatellites with *An. darlingi* populations from northern and southern Amazonian riversides.^46^ Here, we showed a significant *F*_st_ differentiation based on the *per* gene between the Unini and Jaú populations. Although this was the less distant pair of localities evaluated (50 km), they are communities in distinct riversides.

We added *An. darlingi* molecular data important for population genetic analyses and polymorphisms in behavioural and insecticide resistance-related genes. These data can contribute to the discussion if *An. darlingi* is or not a complex of cryptic species, in addition to previous and future studies with a broader sampling, considering both geographic and behavioural parameters. Understanding species complex dynamics has a great epidemiologic significance once they may present distinct vector capacity and different responses to control strategies.^67^

*In conclusion* - Our results add genetic data about differentiation among *An. darlingi* populations, considering *per*, *tim*, and *ace-1* genes*.* These results alone are not enough to support the hypothesis about the existence of cryptic species in *An. darlingi*, however highlights genetic differences between Brazilian and Colombian populations. New non-synonymous variations were identified in the *ace-1* gene and the classical *kdr* mutation was not observed in the *Na*_*V*_ gene.
