# Supplementary material for: Molecular diversity of genes related to biological rhythms (period and timeless) and insecticide resistance (Na V and ace-1) in Anopheles darlingi
Source: Mem Inst Oswaldo Cruz. 2023 Jul 10;118:e220159. doi: 10.1590/0074-02760220159 (PMC10337825; doi:10.1590/0074-02760220159)
Supplement: Supplementary file 1 [file 1678-8060-mioc-118-e220159-s.pdf]

TABLE I

Annealing temperature to different polymerase kits and populations

Gene	Primers	a (ºC)	Population	Reference
per	138f 5'ATGCTGACCAGATTACCGCC3'	69	Manaus and Rondônia	This study
	712r 5'TGAGTTCCTTGCCCTTGTCG3'	57.9	Unini River, Jaú River, Colombia	
tim	*5dartim2a* 5'GAACACAGGTCGAGAAGGAATC3'	63	Manaus and Rondônia	Bottino, 2007[86]
	3dartim3 5'CGTATCGATTTGCACTTGCT3'	52	Unini River	
		56	Jaú River	
		54	Colombia	
Na_v	42f 5'TCGTGTTTTATGCGGAGAATG3'	64	Manaus and Rondônia	This study
	422R 5'CACGGACGCAATTTGACTTGT3'	60	Unini River	
		60	Jaú River	
		60	Colombia	
ace-1	*Adace1F* 5'GCCGGCCGAAAAGTGGAG3'	65	Manaus and Rondônia	This study
	Adace1R 5'CTTAGAGCAAGGTTCTGATCGA3'	56	Colombia	
		60	Unini River	
		60	Jaú River	

a: annealing temperature

TABLE II

Haplotypes found on *per* gene fragment in five populations studied; acession number available on GenBank

Haplotypes	Localities (sequence number)	Total	Acession number
per-1	Unini River (6), Porto Velho (1), Manaus (1)	8	MW415311
per-2	Unini River (1)	1	MW415312
per-3	Unini River (1)	1	MW415313
per-4	Unini River (1), Jaú River (8)	9	MW415314
per-5	Unini River (1), Jaú River (1)	2	MW415315
per-6	Unini River (1)	1	MW415316
per-7	Unini River (1)	1	MW415317
per-8	Unini River (1)	1	MW415318
per-9	Unini River (1)	1	MW415319
per-10	Unini River (1), Porto Velho (1)	2	MW415320
per-11	Unini River (1), Manaus (1)	2	MW415321
per-12	Unini River (1)	1	MW415322
per-13	Jaú River (2)	2	MW415323
per-14	Jaú River (1)	1	MW415324
per-15	Jaú River (1)	1	MW415325
per-16	Jaú River (2)	2	MW415326
per-17	Jaú River (2)	2	MW415327
per-18	Jaú River (1)	1	MW415328
per-19	Jaú River (1)	1	MW415329
per-20	Jaú River (1)	1	MW415330
per-21	Jaú River (1), Manaus (1)	2	MW415331
per-22	Colombia (1)	1	MW415332

CC BY

Haplotypes	Localities (sequence number)	Total	Acession number
per-23	Colombia (2)	2	MW415333
per-24	Colombia (7)	7	MW415334
per-25	Colombia (2)	3	MW415335
per-26	Colombia (1)	1	MW415336
per-27	Colombia (2)	2	MW415337
per-28	Colombia (1)	1	MW415338
per-29	Colombia (1)	1	MW415339
per-30	Colombia (1)	1	MW415340
per-31	Colombia (1)	1	MW415341
per-32	Colombia (1)	1	MW415342
per-33	Colombia (1)	1	MW415343
per-34	Colombia (1)	1	MW415344
per-35	Porto Velho (1)	1	MW415345
per-36	Porto Velho (1)	1	MW415346
per-37	Porto Velho (1)	1	MW415347
per-38	Porto Velho (1)	1	MW415348
per-39	Porto Velho (2)	2	MW415349
per-40	Porto Velho (1)	1	MW415350
per-41	Porto Velho (1)	1	MW415351
per-42	Porto Velho (1), Manaus (2)	3	MW415352
per-43	Porto Velho (1)	1	MW415353
per-44	Porto Velho (1)	1	MW415354
per-45	Porto Velho (1)	1	MW415355
per-46	Porto Velho (1)	1	MW415356
per-47	Porto Velho (1)	1	MW415357
per-48	Porto Velho (1)	1	MW415358
per-49	Porto Velho (1)	1	MW415359
per-50	Porto Velho (1)	1	MW415360
per-51	Porto Velho (1)	1	MW415361
per-52	Porto Velho (1)	1	MW415362
per-53	Manaus (2)	2	MW415363
per-54	Manaus (1)	1	MW415364
per-55	Manaus (1)	1	MW415365
per-56	Manaus (1)	1	MW415366
per-57	Manaus (1)	1	MW415367
per-58	Manaus (1)	1	MW415368
per-59	Manaus (1)	1	MW415369
per-60	Manaus (1)	1	MW415370
per-61	Manaus (1)	1	MW415371
per-62	Manaus (1)	1	MW415372
per-63	Manaus (1)	1	MW415373
per-64	Manaus (1)	1	MW415374
per-65	Manaus (1)	1	MW415375

TABLE III

Polymorphic index found in *per*, *tim*, Na_V and *ace-1* at all five populations

	Unini River	Jaú River	Manaus	Porto Velho	Colombia
			per		
N	17	21	19	21	23
H	12	11	17	20	13
S	23	20	18	20	21
HD	0.890	0.852	0.988	0.995	0.897
Π	0.011 (0.000)	0.008 (0.000)	0.011 (0.000)	0.010 (0.000)	0.007 (0.000)
ϴ	0.012 (0.000)	0.0103 (0.000)	0.009 (0.000)	0.010 (0.000)	0.010 (0.000)
			tim		
N	16	21	15	16	18
H	10	8	9	10	12
S	10	8	10	8	18
HD	0.925	0.757	0.800	0.933	0.922
Π	0.003 (0.000)	0.002 (0.000)	0.003 (0.000)	0.003 (0.000)	0.006 (0.000)
ϴ	0.004 (0.000)	0.003 (0.000)	0.004 (0.000)	0.003 (0.000)	0.007 (0.000)
			Na_V		
N	22	22	19	14	66
H	5	4	4	2	7
S	4	3	3	1	6
HD	0.407	0.260	0.520	0.143	0.175
Π	0.001 (0.000)	0.000 (0.000)	0.001 (0.000)	0.000 (0.000)	0.000 (0)
ϴ	0.003 (0.000)	0.002 (0.000)	0.002 (0.000)	0.000 (0.000)	0.003 (0.000)
			ace-1		
N	16	24	18	17	24
H	8	13	10	12	12
S	11	11	11	11	8
HD	0.758	0.895	0.850	0.919	0.880
Π	0.006 (0.000)	0.007 (0.000)	0.006 (0.000)	0.007 (0.000)	0.006 (0.000)
ϴ	0.009 (0.000)	0.008 (0.000)	0.009 (0.000)	0.009 (0.000)	0.006 (0.000)

N: number of sequences; H: number of haplotypes; S: number of polymorphic sites; Π: pairwise difference mean; ϴ: neutral parameter of segregation sites; Π and ϴ variance are shown between parenthesis.

TABLE IV

Haplotypes found on *tim* fragment in five populations studied; acession number available on GenBank

Haplotypes	Localities (sequence number)	Total	Acession number
tim-1	Unini River (4), Jaú River (10), Manaus (7), Porto Velho (2)	23	MW415376
tim-2	Unini River (2), Jaú River (3), Porto Velho (2)	7	MW415377
tim-3	Unini River (1)	1	MW415378
tim-4	Unini River (2), Manaus (1), Porto Velho (1)	4	MW415379
tim-5	Jaú River (2)	2	MW415380
tim-6	Unini River (1), Jaú River (3), Porto Velho (3)	7	MW415381
tim-7	Unini River (1)	1	MW415382
tim-8	Unini River (2), Manaus (1), Porto Velho (3)	6	MW415383
tim-9	Unini River (1)	1	MW415384
tim-10	Unini River (1)	1	MW415385
tim-11	Jaú River (1), Porto Velho (1)	2	MW415386
tim-12	Jaú River (1), Manaus (1)	2	MW415387
tim-13	Jaú River (1)	1	MW415388
tim-14	Jaú River (1)	1	MW415389
tim-15	Colombia (4)	4	MW415390
tim-16	Colombia (4)	4	MW415391
tim-17	Colombia (1)	1	MW415392
tim-18	Colombia (1)	1	MW415393
tim-19	Colombia (1)	1	MW415394
tim-20	Colombia (1)	1	MW415395
tim-21	Colombia (1)	1	MW415396
tim-22	Colombia (1)	1	MW415397
tim-23	Colombia (1)	1	MW415398
tim-24	Colombia (1)	1	MW415399
tim-25	Colombia (1)	1	MW415400
tim-26	Colombia (1)	1	MW415401
tim-27	Manaus (1)	1	MW415402
tim-28	Manaus (1)	1	MW415403
tim-29	Manaus (1)	1	MW415404
tim-30	Manaus (1), Porto Velho (1)	2	MW415405
tim-31	Manaus (1)	1	MW415406
tim-32	Porto Velho (1)	1	MW415407
tim-33	Porto Velho (1)	1	MW415408
tim-34	Porto Velho (1)	1	MW415409

TABLE V

Haplotypes found on Na_V gene fragment in five populations studied; acession number available on GenBank

Haplotypes	Localities (sequence number)	Total	Acession number
Na_v-1	Unini River (17), Jaú River (19), Manaus (13), Porto Velho (13), Colombia (60)	122	MW388989
Na_v-2	Colombia (1)	1	MW388990
Na_v-3	Colombia (1)	1	MW388991
Na_v-4	Colombia (1)	1	MW388992
Na_v-5	Colombia (1)	1	MW388993
Na_v-6	Colombia (1)	1	MW388994
Na_v-7	Colombia (1)	1	MW388995
Na_v-8	Manaus (2)	2	MW388996
Na_v-9	Jaú River (2), Manaus (3)	5	MW388997
Na_v-10	Manaus (1)	1	MW388998
Na_v-11	Porto Velho (1)	1	MW388999
Na_v-12	Jaú River (1)	1	MW389000
Na_v-13	Jaú River (1)	1	MW389001
Na_v-14	Jaú River (1)	1	MW389002
Na_v-15	Unini River (1)	1	MW389003
Na_v-16	Unini River (1)	1	MW389004
Na_v-17	Unini River (1)	1	MW389005

TABLE VI

Haplotypes found on *ace-1* gene fragment in five populations studied; acession number available on GenBank

Haplotypes	Localities (sequence number)	Total	Acession number
ace-1	Jaú River (1), Manaus (2)	3	MW415275
ace-2	Jaú River (2),Colombia (4), Porto Velho (2)	8	MW415276
ace-3	Unini River (1), Jaú River (1)	2	MW415277
ace-4	Jaú River (2), Manaus (1)	3	MW415278
ace-5	Jaú River (7), Colombia (1), Unini River (8), Manaus (7), Porto Velho (5)	28	MW415279
ace-6	Jaú River (1)	1	MW415280
ace-7	Jaú River (4), Manaus (2)	6	MW415281
ace-8	Jaú River (1), Porto Velho (1)	2	MW415282
ace-9	Jaú River (1), Colombia (1), Manaus (1), Porto Velho (1)	4	MW415283
ace-10	Jaú River (1)	1	MW415284
ace-11	Jaú River (1),Colombia (1)	2	MW415285
ace-12	Jaú River (1)	1	MW415286
ace-13	Jaú River (1)	1	MW415287
ace-14	Colombia (4)	4	MW415288
ace-15	Colombia (7), Porto Velho (1)	8	MW415289
ace-16	Colombia (1)	1	MW415290
ace-17	Colombia (1)	1	MW415291
ace-18	Colombia (1)	1	MW415292
ace-19	Colombia (1)	1	MW415293
ace-20	Colombia (1)	1	MW415294
ace-21	Colombia (1), Manaus (1), Porto Velho (1)	3	MW415295

Haplotypes	Localities (sequence number)	Total	Acession number
ace-22	Unini River (1)	1	MW415296
ace-23	Unini River (2)	2	MW415297
ace-24	Unini River (1)	1	MW415298
ace-25	Unini River (1)	1	MW415299
ace-26	Unini River (1)	1	MW415300
ace-27	Unini River (1)	1	MW415301
ace-28	Manaus (1), Porto Velho (1)	2	MW415302
ace-29	Manaus (1)	1	MW415303
ace-30	Manaus (1)	1	MW415304
ace-31	Manaus (1)	1	MW415305
ace-32	Porto Velho (1)	1	MW415306
ace-33	Porto Velho (1)	1	MW415307
ace-34	Porto Velho (1)	1	MW415308
ace-35	Porto Velho (1)	1	MW415309
ace-36	Porto Velho (1)	1	MW415310

TABLE VII

Neutrality test of four genes in Amazonian populations of *Anopheles darlingi*

			DT					
Population	***DT per***	*p*	***DT tim***	*p*	***DT*** $\boldsymbol{Na_v}$	*p*	***DT ace-1***	*p*
Unini River	-0.316	Ns	-0.511	Ns	-1.478	Ns	-1.365	Ns
Jaú River	-0.641	Ns	-0.490	Ns	-1.729	Ns	-0.345	Ns
Manaus	0.775	Ns	-0.906	Ns	-0.866	Ns	-1.031	Ns
Porto Velho	0.092	Ns	-0.078	Ns	-1.155	Ns	-0.909	Ns
Colombia	-1.195	Ns	-0.258	Ns	-2.065	***	0.313	Ns
			Fs					
	Fs per	*p*	***Fs tim***	*p*	***Fs*** $\boldsymbol{Na_v}$	*p*	***Fs ace-1***	*p*
Unini River	-2.740	ns	-4.428	ns	-2.756	ns	-2.652	ns
Jaú River	-1.816	ns	-2.263	ns	-2.889	ns	-6.261	ns
Manaus	-9.684	ns	-3.797	ns	-1.264	ns	-4.412	ns
Porto Velho	-15.822	ns	-4.900	ns	-0,595	ns	-7.688	ns
Colombia	-4.239	ns	-3.416	ns	-9.173	ns	-5.586	ns
			R2					
	R2 per	*p*	**R2 *tim***	*p*	***R2*** $\boldsymbol{Na_v}$	*p*	***R2 ace-1***	*p*
Unini River	0.13902	ns	0.15029	ns	0.16385	ns	0.15499	ns
Jaú River	0.13276	ns	0.14104	ns	0.16926	ns	0.13262	ns
Manaus	0.13233	ns	0.15392	ns	0.17194	ns	0.14705	ns
Porto Velho	0.12955	ns	0.15227	ns	0.20750	ns	0.14725	ns
Colombia	0.12839	ns	0.13877	ns	0.12216	ns	0.13278	ns

DT: Tajima's test statistics (1989); Ns: non-significant ($p > 0.05$); *significant ($p < 0.05$); ***strongly significant ($p < 0.001$); Fs: Fu's test statistics (1997); ns: non-significant after Bonferroni's correction ($\alpha > 0,01$); R2: Ramos-Onsins and Rosas test statistics (2002).

TABLE VIII

Genetic differentiation of *per*, *tim*, Na_V, and *ace-1* between Amazonian *Anopheles darlingi* populations

	per		*tim*		Na_V		*ace-1*	
	F_{st}	*P*	F_{st}	*P*	F_{st}	*P*	F_{st}	*P*
Uni x Jaú	0.190	***	0	Ns	0.031	Ns	0.047	Ns
Uni x Man	0.051	*	0	Ns	0	Ns	0.022	Ns
Unix Pve	0.033	Ns	0	Ns	0.037	Ns	0.164	***
Jaú x Man	0.187	***	0	Ns	0.051	**	0	Ns
Jaú x Pve	0.178	***	0.014	Ns	0	Ns	0.047	Ns
Man x Pve	0.027	Ns	0	Ns	0.060	Ns	0.069	Ns
Col x Uni	0.220	***	0.347	***	0.035	**	0.330	***
Col x Jaú	0.475	***	0.389	***	0	Ns	0.209	***
Col x Man	0.318	***	0.374	***	0.057	*	0.255	***
Col x Pve	0.208	***	0.354	***	0	Ns	0.054	*

Fst: pairwise fixation index; P: significance of Fst estimative with 1000 random permutations; ***p < 0.001; **p > 0.01; *p > 0.05; ns: non-significant; Nm: migrant number; Uni: Unini River; Jaú: Jaú River; Man: Manaus; Pve: Porto Velho; Col: Colombia. In shade comparison between Brazil and Colombia.

TABLE IX

Polymorphism index at five populations

	Ss	*Sf*	*Sx*	*Sy*	*Nm*
		per			
Uni x Jaú	17	0	6	3	1,06
Uni x Man	15	0	8	3	4,61
Unix Pve	17	0	6	3	7,29
Jaú x Man	14	0	6	4	1,08
Jaú x Pve	17	0	3	3	1,15
Man x Pve	16	0	2	4	8,83
Col x Uni	10	0	13	11	0,88
Col x Jaú	11	0	9	10	0,27
Col x Man	9	0	12	9	0,53
Col x Pve	11	0	10	9	0,95
		tim			
Uni x Jaú	6	0	2	4	erro
Uni x Man	8	0	2	2	erro
Uni x Pve	7	0	1	3	erro
Jaú x Man	6	0	4	2	erro
Jaú x Pve	7	0	1	1	17,18
Man x Pve	7	0	1	3	erro
Col x Uni	4	0	6	14	0,47
Col x Jaú	3	0	5	15	0,39
Col x Man	4	0	6	14	0,42
Col x Pve	3	0	5	15	0,46

	Ss	*Sf*	*Sx*	*Sy*	*Nm*
			Na_V		
Uni x Jaú	0	0	3	4	7,63
Uni x Man	1	0	2	3	erro
Uni x Pve	0	0	1	4	6
Jaú x Man	0	0	3	3	5
Jaú x Pve	0	0	1	3	erro
Man x Pve	0	0	1	3	4
Col x Uni	0	0	4	6	7
Col x Jaú	0	0	3	6	erro
Col x Man	0	0	3	6	4
Col x Pve	0	0	1	6	erro
			ace-1		
Uni x Jaú	5	0	6	6	4,99
Uni x Man	5	0	6	6	10,69
Uni x Pve	3	0	8	8	1,27
Jaú x Man	9	0	2	2	erro
Jaú x Pve	6	0	5	5	5,06
Man x Pve	7	0	4	4	3,35
Col x Uni	3	0	5	8	0,51
Col x Jaú	4	0	7	4	0,94
Col x Man	5	0	6	3	0,73
Col x Pve	5	0	6	3	4,35

Ss: pairwise shared polymorphism amount; Sf: pairwise fixed substitution amount; Sx and Sy: exclusive pairwise polymorphism amount of population 1 and 2 respectively; Nm: number of migrants per generation; Uni: Unini River; Jaú: Jaú River; Man: Manaus; Pve: Porto Velho; Col: Colombia. In shade comparison between Brazil and Colombia.

TABLE X

Mantel test

	All populations		Brazilian populations	
Gene	R	P	R	P
per	0.3777	ns	(-0.5603)	ns
tim	0.7154	*	0.3652	ns
ace-1	0.5998	ns(*)	0.4270	ns
Na_V	(-0.0551)	ns	0.0522	ns

R: correlation coefficient; ns: $p > 0.05$; *$p < 0.05$. Significance testing with 10000 permutations.

TABLE XI

Correlation between geographic [Ln (km) and genetic distances (F*st*/(1-F*st*)]

		Fst/(1-Fst)			
Pair of populations	Ln (km)	*per*	*tim*	*ace-1*	*Na_V*
Uni x Jaú	3.97	0.2346	0.0000	0.0493	0.0320
Uni x Col	7.50	0.2821	0.5314	0.4925	0.0363
Uni x Pve	6.70	0.0341	0.0000	0.1962	0.0384
Uni x Man	5.68	0.0537	0.0000	0.0225	0.0000
Jaú x Col	7.53	0.9048	0.6367	0.2642	0.0000
Jaú x Pve	6.74	0.2165	0.0142	0.0493	0.0000
Jaú x Man	5.54	0.2300	0.0000	0.0000	0.0537
Col x Pve	7.50	0.2626	0.5480	0.0571	0.0000
Col x Man	7.65	0.4663	0.5974	0.3423	0.0604
Pve x Man	6.85	0.0277	0.0000	0.0741	0.0638

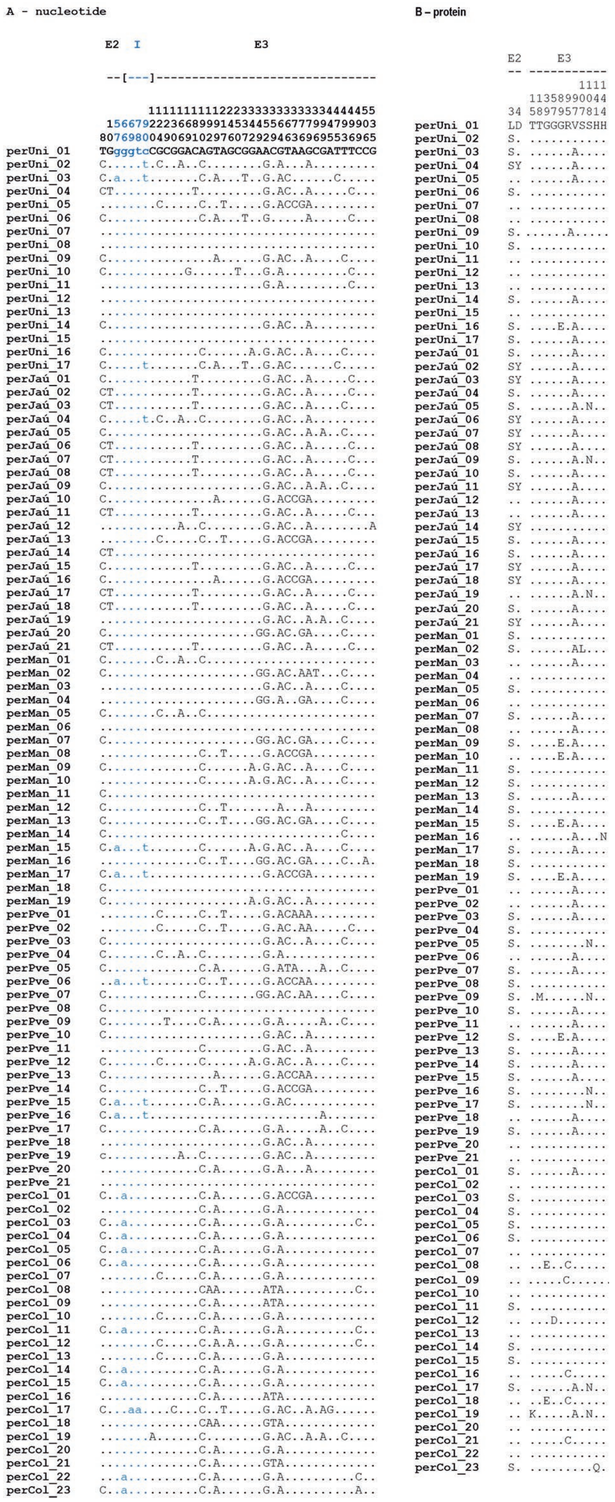

Fig. 1: *per* fragment alignment. The alignment represents the polymorphic sites amongst sequences from five populations. Numbers in the header consist of polymorphic site position in the alignment of nucleotides (A) and predicted amino acids (B). Dots indicate identity to the first sequence. E: exon. I: intron. Uni: Unini River; Jaú: Jaú River; Col: Colombia; Pve: Porto Velho; Man: Manaus.

A – nucleotide

```
                         E3      I E4      I

                 --------------------------

                    1111122222344445555556677
                 2368225681345814551455996702
                 9745374550710913040949389963
timUni 01        AGGTAGGGtgcccTccgctccggagggg
timUni_02        .............G.......c......
timUni 03        .A..........................
timUni_04        .............Gg.........a...
timUni_05        ..AC..........g.............
timUni_06        .............G.......c......
timUni_07        .............Gg.........a...
timUni_08        .............Gg.............
timUni_09        ..AC.........Gg.............
timUni_10        ...........t.Gg.............
timUni_11        ................t...........
timUni_12        ............................
timUni_13        ..........................a.
timUni_14        ...........t.Gg.............
timUni_15        ............................
timUni_16        ............................
timJaú_01        ............................
timJaú_02        ............................
timJaú_03        .....A......................
timJaú_04        .............G.......c......
timJaú_05        ............................
timJaú_06        .............Gg.............
timJaú_07        ............................
timJaú_08        .............Gg.............
timJaú_09        .............G.......c......
timJaú_10        ............................
timJaú_11        ............................
timJaú_12        .............Gg......c......
timJaú_13        ..AC..........g.............
timJaú_14        ..AC........................
timJaú_15        .............Gg...c.....a...
timJaú_16        ............................
timJaú_17        ............................
timJaú_18        ............................
timJaú_19        .............G.......c......
timJaú_20        ............................
timJaú_21        .............Gg.............
timMan 01        .............Gg......c......
timMan 02        ............................
timMan 03        .............Gg.........a...
timMan 04        ............................
timMan 05        ..AC..........g........g....
timMan 06        ...........t.Gg.............
timMan 07        ............................
timMan 08        ............................
timMan 09        ..............g.............
timMan 10        ............................
timMan 11        ............................
timMan 12        ............................
timMan 13        ..A..........Gg...........a.
timMan 14        .............G..............
timMan 15        ........a....Gg...........a.
timPve 01        .............Gg.............
timPve 02        ...........t................
timPve 03        .............Gg.........a...
timPve 04        ..AC.................c......
timPve 05        ............................
timPve 06        .............Gg.............
timPve 07        .............Gg.............
timPve 08        .....A......................
timPve 09        .............G.......c......
timPve 10        .............G.......c......
timPve 11        .............G..............
timPve 12        ...........t.Gg.............
timPve 13        ...C........................
timPve 14        ...........t.Gg.............
timPve 15        ............................
timPve 16        ...........t.Gg.............
timCol 01        ...C.....at..G.t..........c..
timCol 02        ...C.....at..G.t..........c..
timCol 03        ...C.........G...t..g.....a.
timCol 04        ...C.........G...t..g.....a.
timCol 05        ...C.....at.tG.t..........c.a
timCol 06        ...C.....at..G.t..........c..
timCol 07        ..AC......t..Gg..t..........
timCol 08        ......A......G..............
timCol 09        ...C.....at..G.t......t..c..
timCol 10        ...C.........G...........c..
timCol 11        ..AC.........G...t..g.....a.
timCol 12        ...CG........G...........c..
timCol 13        ...C...A.....G...t..g....c..
timCol 14        ...C.....at..G.t..........c..
timCol 15        ...C.........G.....a.....c..
timCol 16        G..C.........Gg..t..g.....a.
timCol 17        ...C.........G...t..g.....a.
timCol 18        ...C.........G...t..g.....a.
```

B – protein

```
                 E3
                 ---
                 145
                 015
timUni_01        TQG
timUni_02        ...
timUni_03        ...
timUni_04        ...
timUni_05        ...
timUni_06        ...
timUni_07        ...
timUni_08        ...
timUni_09        ...
timUni_10        ...
timUni_11        ...
timUni_12        ...
timUni_13        ...
timUni_14        ...
timUni_15        ...
timUni_16        ...
timJaú_01        ...
timJaú_02        ...
timJaú_03        ...
timJaú_04        ...
timJaú_05        ...
timJaú_06        ...
timJaú_07        ...
timJaú_08        ...
timJaú_09        ...
timJaú_10        ...
timJaú_11        ...
timJaú_12        ...
timJaú_13        ...
timJaú_14        ...
timJaú_15        ...
timJaú_16        ...
timJaú_17        ...
timJaú_18        ...
timJaú_19        ...
timJaú_20        ...
timJaú_21        ...
timMan 01        ...
timMan 02        ...
timMan 03        ...
timMan 04        ...
timMan 05        ...
timMan 06        ...
timMan 07        ...
timMan 08        ...
timMan 09        ...
timMan 10        ...
timMan 11        ...
timMan 12        ...
timMan 13        ...
timMan 14        ...
timMan 15        ...
timPve 01        ...
timPve 02        ...
timPve 03        ...
timPve 04        ...
timPve 05        ...
timPve 06        ...
timPve 07        ...
timPve 08        ...
timPve 09        ...
timPve 10        ...
timPve 11        ...
timPve 12        ...
timPve 13        ...
timPve 14        ...
timPve 15        ...
timPve 16        ...
timCol 01        ...
timCol 02        ...
timCol 03        ...
timCol 04        ...
timCol 05        ...
timCol 06        ...
timCol 07        ...
timCol 08        ...
timCol 09        ...
timCol 10        ...
timCol 11        ...
timCol 12        .R.
timCol 13        ..D
timCol 14        ...
timCol 15        ...
timCol 16        A..
timCol 17        ...
timCol 18        ...
```

Fig. 2: *tim* fragment alignment. The alignment represents the polymorphic sites amongst sequences from five populations. Numbers in the header consist of polymorphic site position in the alignment of nucleotides (A) and predicted amino acids (B). Dots indicate identity to the first sequence. E: exon. I: intron. Uni: Unini River; Jaú: Jaú River; Col: Colombia; Pve: Porto Velho; Man: Manaus.

A – nucleotide

```
              E20  I   E21
             ----------------

                  11112222223
             1345823493577891
             4081844232527040
NavUni 01    TTTCTataTGATCTAA   NavCol 01    ................
NavUni 02    ................   NavCol 02    ................
NavUni 03    ................   NavCol 03    ..........G.....
NavUni 04    ................   NavCol 04    ................
NavUni 05    ................   NavCol 05    ................
NavUni 06    ..C.............   NavCol 06    ................
NavUni 07    ............C...   NavCol 07    ................
NavUni 08    ....C...........   NavCol 08    ................
NavUni 09    ....C......C....   NavCol 09    ................
NavUni 10    ................   NavCol 10    C...............
NavUni 11    ................   NavCol 11    ................
NavUni 12    ................   NavCol 12    .......g........
NavUni 13    ................   NavCol 13    ................
NavUni 14    ................   NavCol 14    ................
NavUni 15    ................   NavCol 15    ................
NavUni 16    ................   NavCol 16    ................
NavUni 17    ................   NavCol 17    ................
NavUni 18    ....C...........   NavCol 18    ................
NavUni 19    ................   NavCol 19    ................
NavUni 20    ................   NavCol 20    ................
NavUni 21    ................   NavCol 21    ................
NavUni 22    ................   NavCol 22    ................
NavJaú 01    ................   NavCol 23    ................
NavJaú 02    ................   NavCol 24    ................
NavJaú 03    ................   NavCol 25    ................
NavJaú 04    ................   NavCol 26    ................
NavJaú 05    ............T...   NavCol 27    ......c.........
NavJaú 06    ................   NavCol 28    ................
NavJaú 07    ................   NavCol 29    ................
NavJaú 08    ................   NavCol 30    ................
NavJaú 09    ................   NavCol 31    ................
NavJaú 10    ................   NavCol 32    ................
NavJaú 11    ........C.......   NavCol 33    ................
NavJaú 12    ................   NavCol 34    ................
NavJaú 13    ................   NavCol 35    ................
NavJaú 14    ................   NavCol 36    ................
NavJaú 15    ................   NavCol 37    ................
NavJaú 16    ................   NavCol 38    ................
NavJaú 17    ...T............   NavCol 39    ................
NavJaú 18    ................   NavCol 40    ................
NavJaú 19    ................   NavCol 41    ................
NavJaú 20    ................   NavCol 42    ................
NavJaú 21    ................   NavCol 43    ................
NavJaú 22    ................   NavCol 44    ................
NavMan 01    ................   NavCol 45    ................
NavMan 02    .....g..........   NavCol 46    ................
NavMan 03    ................   NavCol 47    ...............G
NavMan 04    ................   NavCol 48    .C..............
NavMan 05    ................   NavCol 49    ................
NavMan 06    .....g..........   NavCol 50    ................
NavMan 07    ................   NavCol 51    ................
NavMan 08    ................   NavCol 52    ................
NavMan 09    ................   NavCol 53    ................
NavMan 10    ....C...........   NavCol 54    ................
NavMan 11    ....C...........   NavCol 55    ................
NavMan 12    .........A......   NavCol 56    ................
NavMan 13    ................   NavCol 57    ................
NavMan 14    ................   NavCol 58    ................
NavMan 15    ................   NavCol 59    ................
NavMan 16    ................   NavCol 60    ................
NavMan 17    ................   NavCol 61    ................
NavMan 18    ....C...........   NavCol 62    ................
NavMan 19    ................   NavCol 63    ................
NavPve 01    ................   NavCol 64    ................
NavPve 02    ................   NavCol 65    ................
NavPve 03    ................   NavCol 66    ................
NavPve 04    ................
NavPve 05    ................
NavPve 06    ................
NavPve 07    ................
NavPve 08    ................
NavPve 09    ..............T.
NavPve 10    ................
NavPve 11    ................
NavPve 12    ................
NavPve 13    ................
NavPve 14    ................
```

B – protein

```
              E20   E21
             ----- ------

              1112 456667
             50678 036899

NavUni 01    WVIPL SDIRFN   NavCol 01    ..... ......
NavUni 02    ..... ......   NavCol 02    ..... ......
NavUni 03    ..... ......   NavCol 03    ..... ......
NavUni 04    ..... ......   NavCol 04    ..... ......
NavUni 05    ..... ......   NavCol 05    ..... ......
NavUni 06    ..T.. ......   NavCol 06    ..... ......
NavUni 07    ..... ....L.   NavCol 07    ..... ......
NavUni 08    ..... ......   NavCol 08    ..... ......
NavUni 09    ..... ..T...   NavCol 09    ..... ......
NavUni 10    ..... ......   NavCol 10    R.... ......
NavUni 11    ..... ......   NavCol 11    ..... ......
NavUni 12    ..... ......   NavCol 12    ..... ......
NavUni 13    ..... ......   NavCol 13    ..... ......
NavUni 14    ..... ......   NavCol 14    ..... ......
NavUni 15    ..... ......   NavCol 15    ..... ......
NavUni 16    ..... ......   NavCol 16    ..... ......
NavUni 17    ..... ......   NavCol 17    ..... ......
NavUni 18    ..... ......   NavCol 18    ..... ......
NavUni 19    ..... ......   NavCol 19    ..... ......
NavUni 20    ..... ......   NavCol 20    ..... ......
NavUni 21    ..... ......   NavCol 21    ..... ......
NavUni 22    ..... ......   NavCol 22    ..... ......
NavJaú 01    ..... ......   NavCol 23    ..... ......
NavJaú 02    ..... ......   NavCol 24    ..... ......
NavJaú 03    ..... ......   NavCol 25    ..... ......
NavJaú 04    ..... ......   NavCol 26    ..... ......
NavJaú 05    ..... ...C..   NavCol 27    ..... ......
NavJaú 06    ..... ......   NavCol 28    ..... ......
NavJaú 07    ..... ......   NavCol 29    ..... ......
NavJaú 08    ..... ......   NavCol 30    ..... ......
NavJaú 09    ..... ......   NavCol 31    ..... ......
NavJaú 10    ..... ......   NavCol 32    ..... ......
NavJaú 11    ..... P.....   NavCol 33    ..... ......
NavJaú 12    ..... ......   NavCol 34    ..... ......
NavJaú 13    ..... ......   NavCol 35    ..... ......
NavJaú 14    ..... ......   NavCol 36    ..... ......
NavJaú 15    ..... ......   NavCol 37    ..... ......
NavJaú 16    ..... ......   NavCol 38    ..... ......
NavJaú 17    ...L. ......   NavCol 39    ..... ......
NavJaú 18    ..... ......   NavCol 40    ..... ......
NavJaú 19    ..... ......   NavCol 41    ..... ......
NavJaú 20    ..... ......   NavCol 42    ..... ......
NavJaú 21    ..... ......   NavCol 43    ..... ......
NavJaú 22    ..... ......   NavCol 44    ..... ......
NavMan 01    ..... ......   NavCol 45    ..... ......
NavMan 02    ..... ......   NavCol 46    ..... ......
NavMan 03    ..... ......   NavCol 47    ..... .....D
NavMan 04    ..... ......   NavCol 48    .A... ......
NavMan 05    ..... ......   NavCol 49    ..... ......
NavMan 06    ..... ......   NavCol 50    ..... ......
NavMan 07    ..... ......   NavCol 51    ..... ......
NavMan 08    ..... ......   NavCol 52    ..... ......
NavMan 09    ..... ......   NavCol 53    ..... ......
NavMan 10    ..... ......   NavCol 54    ..... ......
NavMan 11    ..... ......   NavCol 55    ..... ......
NavMan 12    ..... .N....   NavCol 56    ..... ......
NavMan 13    ..... ......   NavCol 57    ..... ......
NavMan 14    ..... ......   NavCol 58    ..... ......
NavMan 15    ..... ......   NavCol 59    ..... ......
NavMan 16    ..... ......   NavCol 60    ..... ......
NavMan 17    ..... ......   NavCol 61    ..... ......
NavMan 18    ..... ......   NavCol 62    ..... ......
NavMan 19    ..... ......   NavCol 63    ..... ......
NavPve 01    ..... ......   NavCol 64    ..... ......
NavPve 02    ..... ......   NavCol 65    ..... ......
NavPve 03    ..... ......   NavCol 66    ..... ......
NavPve 04    ..... ......
NavPve 05    ..... ......
NavPve 06    ..... ......
NavPve 07    ..... ......
NavPve 08    ..... ......
NavPve 09    ..... ......
NavPve 10    ..... ......
NavPve 11    ..... ......
NavPve 12    ..... ......
NavPve 13    ..... ......
NavPve 14    ..... ......
```

Fig. 3: Na_V fragment alignment. The alignment represents the polymorphic sites amongst sequences from five populations. Numbers in the header consist of polymorphic site position in the alignment of nucleotides (A) and predicted amino acids (B). Dots indicate identity to the first sequence. E: exon. I: intron. Uni: Unini River; Jaú: Jaú River; Col: Colombia; Pve: Porto Velho; Man: Manaus. In red: classic *kdr* mutation site with leucine amino acid related to wild allele.

```
A - nucleotide

                  E5
             -------------------------
               11244682346668891357782
               11244682346668891357782
             5709569441650491737591461
ace1Jaú 01   GAGCGCCTTCGGCGCCGGCAAGACA
ace1Jaú 02   ...GA....GA........G.....
ace1Jaú 03   ...GA..............G.....
ace1Jaú 04   ...GA....G.....T..TG.....
ace1Jaú 05   ...G...............G.....
ace1Jaú 06   ...GA....G.....T..TG.....
ace1Jaú 07   .........G.....T..TG.....
ace1Jaú 08   ...G...............G.....
ace1Jaú 09   ...................G.....
ace1Jaú 10   ...G.....G......A..G.....
ace1Jaú 11   ...GA....GA........G.....
ace1Jaú 12   ...GA....G.........G.....
ace1Jaú 13   ...................G.....
ace1Jaú 14   A..G.......A.......G.....
ace1Jaú 15   ...................G.....
ace1Jaú 16   ...G...............G.....
ace1Jaú 17   ...G...............G.....
ace1Jaú 18   ...................G.....
ace1Jaú 19   ...G.....GA........G.....
ace1Jaú 20   ...G...............G.....
ace1Jaú 21   A..G.......A.......G....T
ace1Jaú 22   ...G...............G.....
ace1Jaú 23   ...G...............G.....
ace1Jaú 24   ...G.......A....A..G.....
ace1Uni 01   ...G...............G.....
ace1Uni 02   ...GA..............G.....
ace1Uni 03   .G.G...............G..GT.
ace1Uni 04   ...G.....G.....T..TG.....
ace1Uni 05   .G.G...............G.....
ace1Uni 06   ..AG...............G.....
ace1Uni 07   ...G...............G.....
ace1Uni 08   ...G...............G.....
ace1Uni 09   ...G...............G.....
ace1Uni 10   .G.G....C..........G..GT.
ace1Uni 11   ...G...............G.....
ace1Uni 12   ...G...............G.....
ace1Uni 13   ...G.....G.....T..TG.....
ace1Uni 14   ...G...............G.....
ace1Uni 15   ...G...............G.....
ace1Uni 16   .............T.....G.....
ace1Man 01   ...G...............G.....
ace1Man 02   ...................G.....
ace1Man 03   ...G...............G.....
ace1Man 04   ...G.....................
ace1Man 05   ...G..............TG.C...
ace1Man 06   ...GA....G.........G.....
ace1Man 07   .........................
ace1Man 08   ...GA....G.....T..TG.....
ace1Man 09   ...G.....GA.....A..G.....
ace1Man 10   ...G...............G.....
ace1Man 11   ...G.......A.......G.....
ace1Man 12   ...GA....GA.T......G.....
ace1Man 13   ...G...............G.....
ace1Man 14   .........................
ace1Man 15   ...G...............G.....
ace1Man 16   ...................G.....
ace1Man 17   ...G...............G.....
ace1Man 18   ...G...............G.....

ace1Pve 01   ...G.....G......A..G.....
ace1Pve 02   ...GA....GA........G.....
ace1Pve 03   ...GA....GA.T......G.....
ace1Pve 04   ...G...............G.....
ace1Pve 05   ...GA....GA........G.....
ace1Pve 06   ...G.....................
ace1Pve 07   .........GA........G.....
ace1Pve 08   ...G...............G.....
ace1Pve 09   ...G.....G....T..C.G.....
ace1Pve 10   ...GAA...GA........G.....
ace1Pve 11   ...G...C.G.........G.....
ace1Pve 12   ...GA....G.........G.....
ace1Pve 13   ...G...............G.....
ace1Pve 14   ...G...............G.....
ace1Pve 15   ...G...............G.....
ace1Pve 16   ...G.....G.........G.....
ace1Pve 17   ...GA....G...............
ace1Col 01   ...G.....GA.T...A..G.....
ace1Col 02   ...GA....G.........G.....
ace1Col 03   ...G.....G.........G.....
ace1Col 04   ...GA....GA........G.....
ace1Col 05   ...G.....GA.T...A..G.....
ace1Col 06   ...G.....G.........G.....
ace1Col 07   ...G.....GA........G.....
ace1Col 08   ...G.....GA.T...A..G.....
ace1Col 09   ...G...............G.....
ace1Col 10   .G.G..G..G.........GG....
ace1Col 11   ...G.....GA.T...A..G.....
ace1Col 12   ...G.....G.........G.....
ace1Col 13   ...G.....G.........G.....
ace1Col 14   ...G.....G.........G.....
ace1Col 15   ...GA....GA........GG....
ace1Col 16   .G.GA....G ........G ....
ace1Col 17   ...G.....G.........G.....
ace1Col 18   ...GA....GA........G.....
ace1Col 19   ...G.....G..T...A..G.....
ace1Col 20   ...G.....G.........G.....
ace1Col 21   .G.G..G..G.........G.....
ace1Col 22   ...GA....GA.T......G.....
ace1Col 23   ...GA....GA........G.....
ace1Col 24   ...GA....GA........G.....
```

```
B - protein

              E5
             -----
                 1
              2560
             28547

ace1Jaú 01   VMAGE
ace1Jaú_02   .....
ace1Jaú_03   .....
ace1Jaú_04   .....
ace1Jaú_05   .....
ace1Jaú_06   .....
ace1Jaú_07   .....
ace1Jaú_08   .....
ace1Jaú_09   .....
ace1Jaú_10   .....
ace1Jaú_11   .....
ace1Jaú_12   .....
ace1Jaú_13   .....
ace1Jaú_14   I....
ace1Jaú_15   .....
ace1Jaú_16   .....
ace1Jaú_17   .....
ace1Jaú_18   .....
ace1Jaú_19   .....
ace1Jaú_20   .....
ace1Jaú_21   I...V
ace1Jaú_22   .....
ace1Jaú_23   .....
ace1Jaú_24   .....
ace1Uni_01   .....
ace1Uni_02   .....
ace1Uni_03   .....
ace1Uni_04   .....
ace1Uni_05   .....
ace1Uni_06   .....
ace1Uni_07   .....
ace1Uni_08   .....
ace1Uni_09   .....
ace1Uni_10   .T...
ace1Uni_11   .....
ace1Uni_12   .....
ace1Uni_13   .....
ace1Uni_14   .....
ace1Uni_15   .....
ace1Uni_16   ..S..
ace1Man 01   .....
ace1Man 02   .....
ace1Man 03   .....
ace1Man 04   .....
ace1Man 05   .....
ace1Man 06   .....
ace1Man 07   .....
ace1Man 08   .....
ace1Man 09   .....
ace1Man 10   .....
ace1Man 11   .....
ace1Man 12   .....
ace1Man 13   .....
ace1Man 14   .....
ace1Man 15   .....
ace1Man 16   .....
ace1Man 17   .....
ace1Man 18   .....

ace1Pve 01   .....
ace1Pve 02   .....
ace1Pve 03   .....
ace1Pve 04   .....
ace1Pve 05   .....
ace1Pve 06   .....
ace1Pve 07   .....
ace1Pve 08   .....
ace1Pve 09   .....
ace1Pve 10   .....
ace1Pve 11   .....
ace1Pve 12   .....
ace1Pve 13   .....
ace1Pve 14   .....
ace1Pve 15   .....
ace1Pve 16   .....
ace1Pve 17   .....
ace1Col 01   .....
ace1Col 02   .....
ace1Col 03   .....
ace1Col 04   .....
ace1Col 05   .....
ace1Col 06   .....
ace1Col 07   .....
ace1Col 08   .....
ace1Col 09   .....
ace1Col 10   .....
ace1Col 11   .....
ace1Col 12   .....
ace1Col 13   .....
ace1Col 14   .....
ace1Col 15   .....
ace1Col 16   .....
ace1Col 17   .....
ace1Col 18   .....
ace1Col 19   .....
ace1Col 20   .....
ace1Col 21   .....
ace1Col 22   .....
ace1Col 23   .....
ace1Col 24   .....
```

Fig. 4: *ace-1* fragment alignment. The alignment represents the polymorphic sites amongst sequences from five populations. Numbers in the header consist of polymorphic site position in the alignment of nucleotides (A) and predicted amino acids (B). Dots indicate identity to the first sequence. E: exon. I: intron. Uni: Unini River; Jaú: Jaú River; Col: Colombia; Pve: Porto Velho; Man: Manaus.

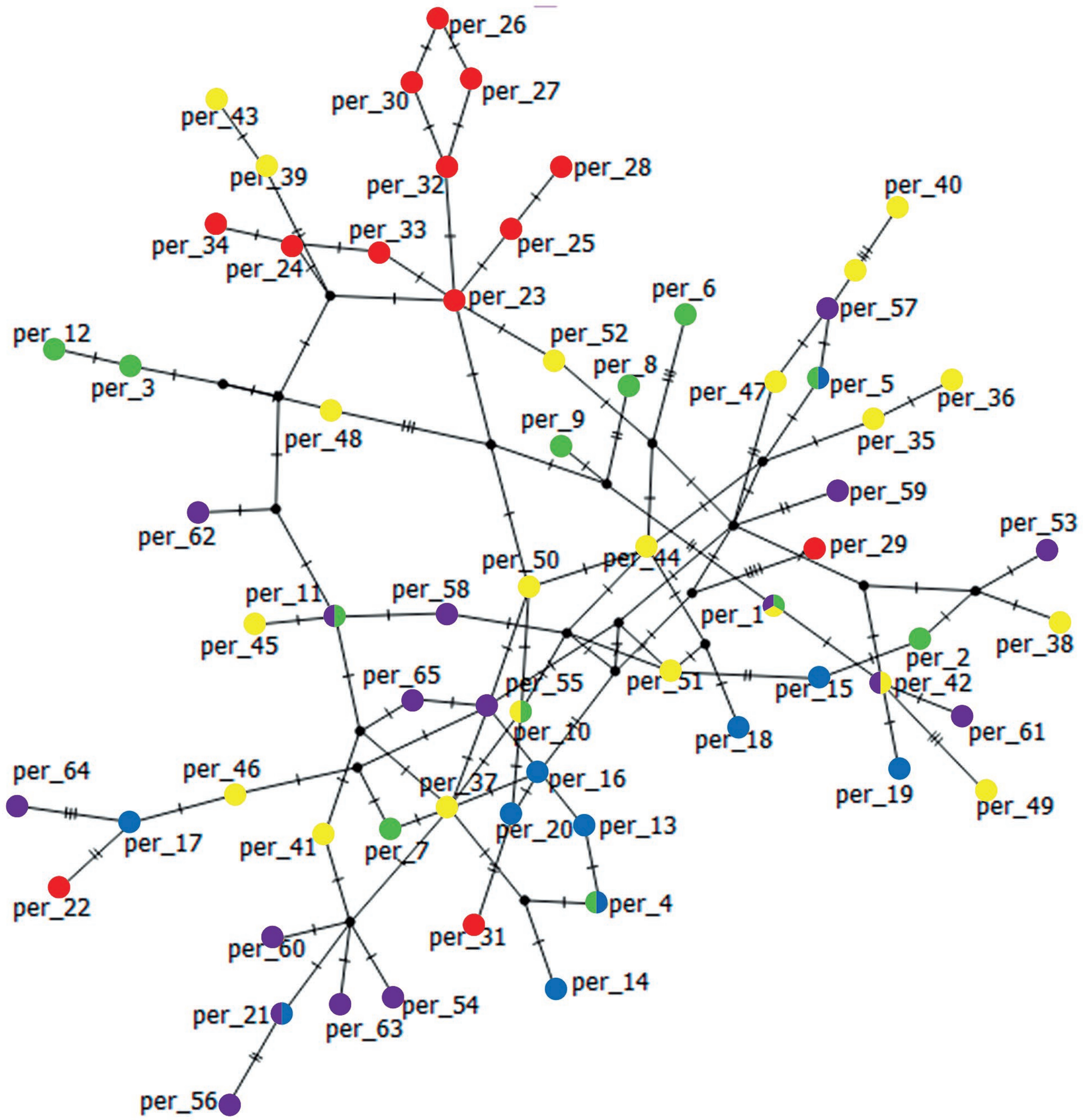

Fig. 5: haplotype network of *per* in *Anopheles darlingi* populations. In green: Unini River, blue: Jaú River, yellow: Porto Velho, purple: Manaus; red: Colombia.

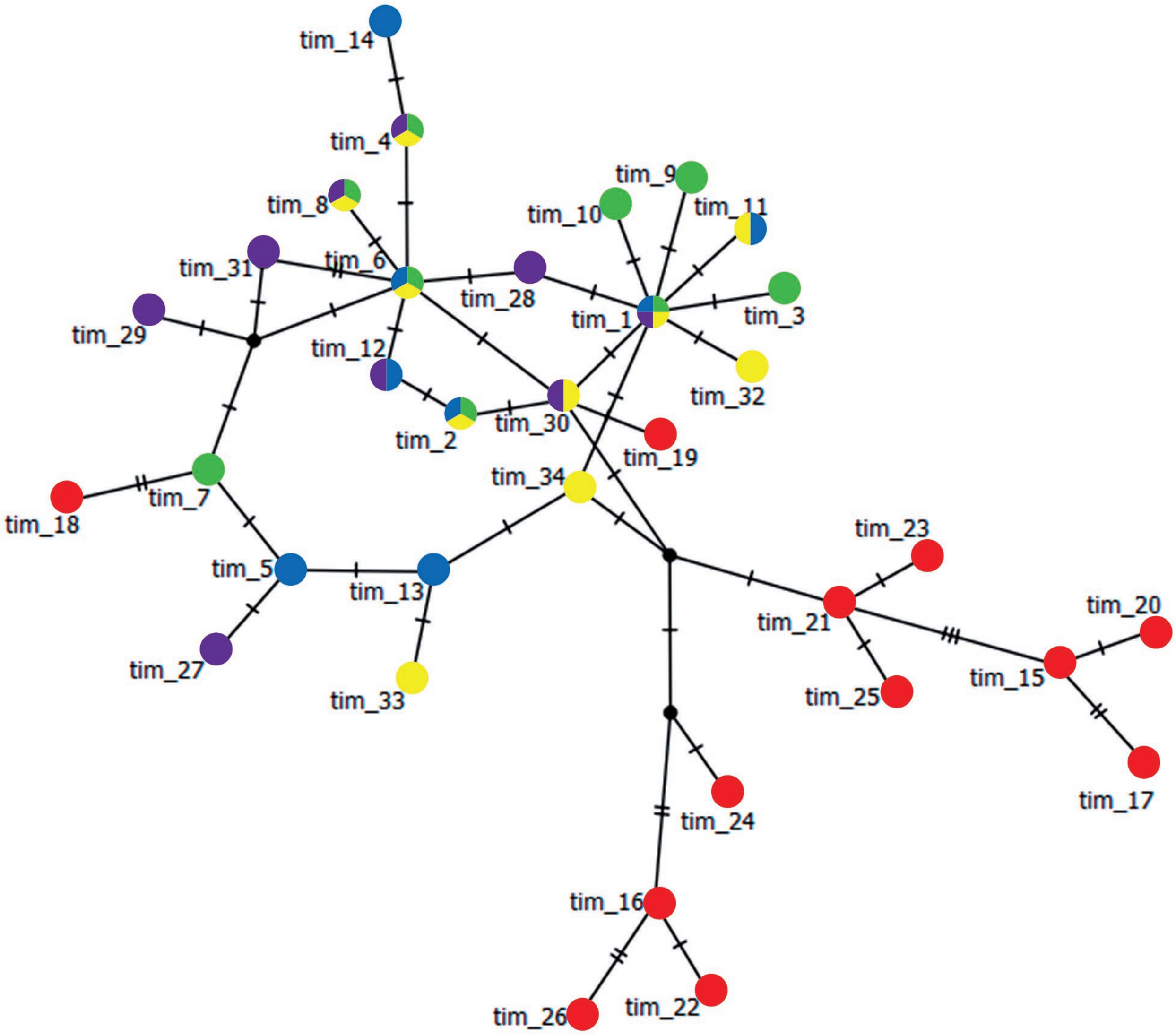

Fig. 6: haplotype network of *tim* in *Anopheles darlingi* populations. In green: Unini River, blue: Jaú River, yellow: Porto Velho, purple: Manaus; red: Colombia.

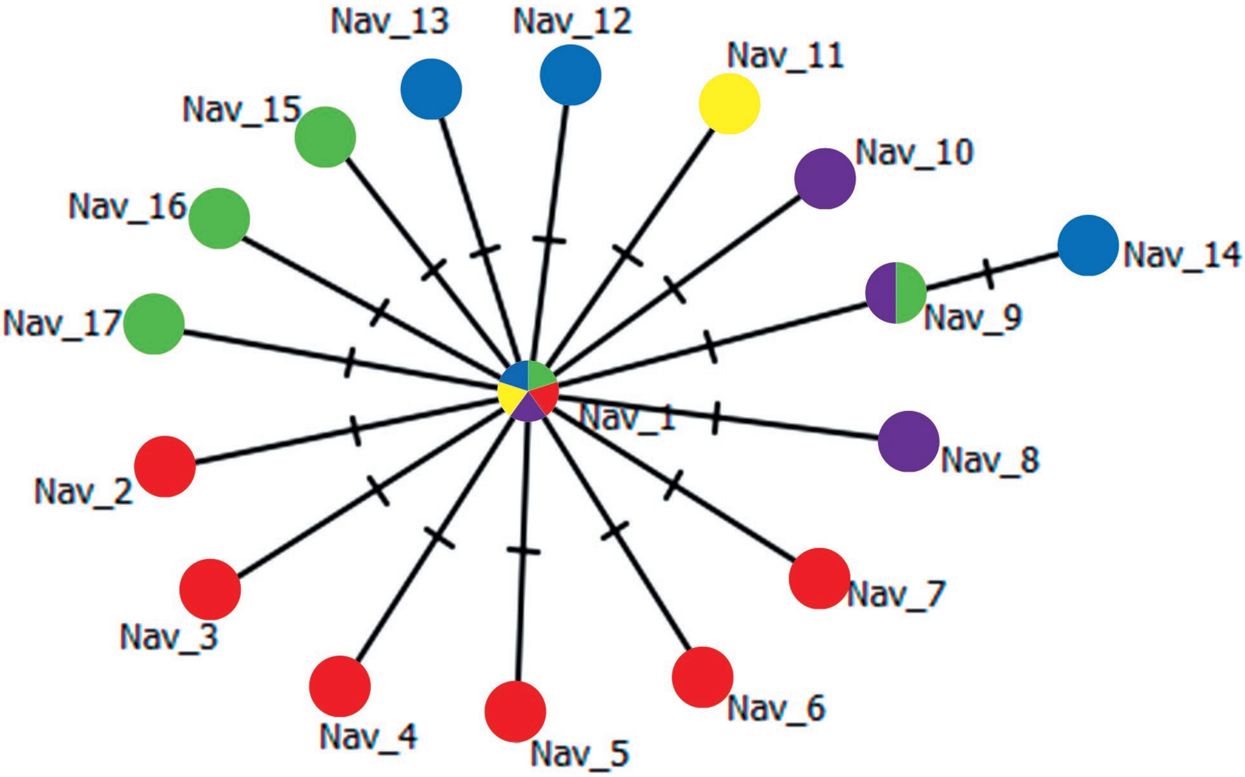

Fig. 7: haplotype network of Na_V in *Anopheles darlingi* populations. In green: Unini River, blue: Jaú River, yellow: Porto Velho, purple: Manaus; red: Colombia.

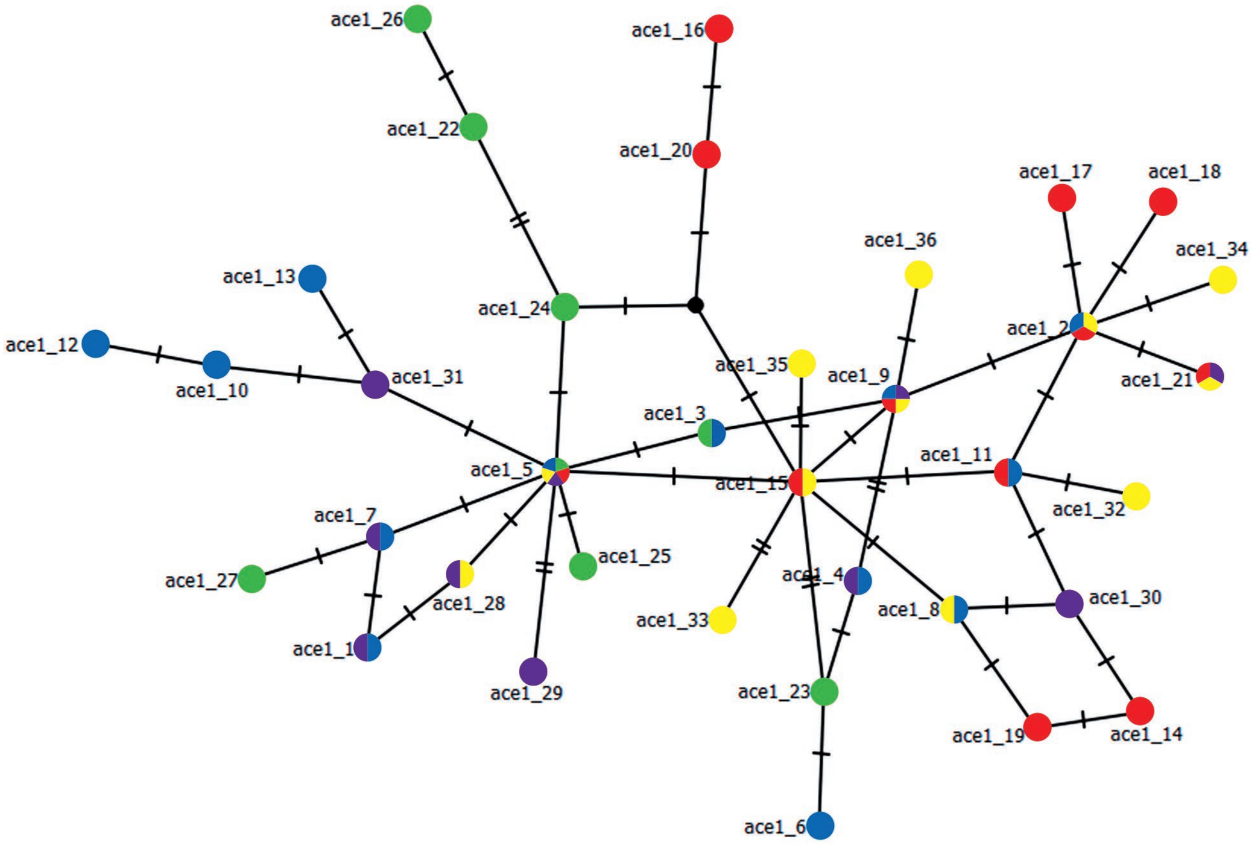

Fig. 8: haplotype network of *ace-1* in *Anopheles darlingi* populations. In green: Unini River, blue: Jaú River, yellow: Porto Velho, purple: Manaus; red: Colombia.

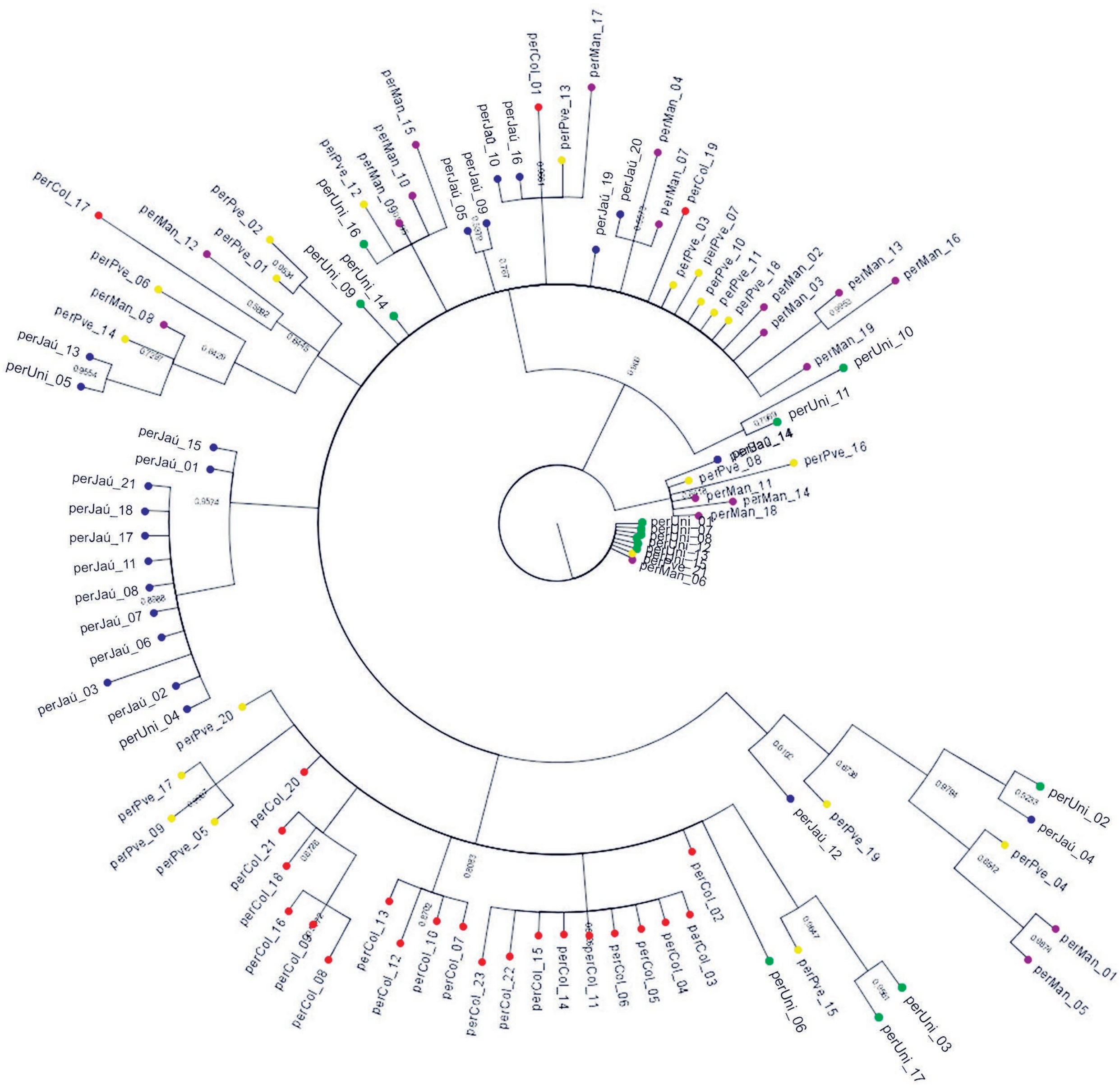

Fig. 9: Bayesian phylogenetic general time reversible tree to *per Anopheles darlingi* populations. In green: Unini River, blue: Jaú River, yellow: Porto Velho, purple: Manaus; red: Colombia. The numbers at the nodes represent the Bayesian posterior.

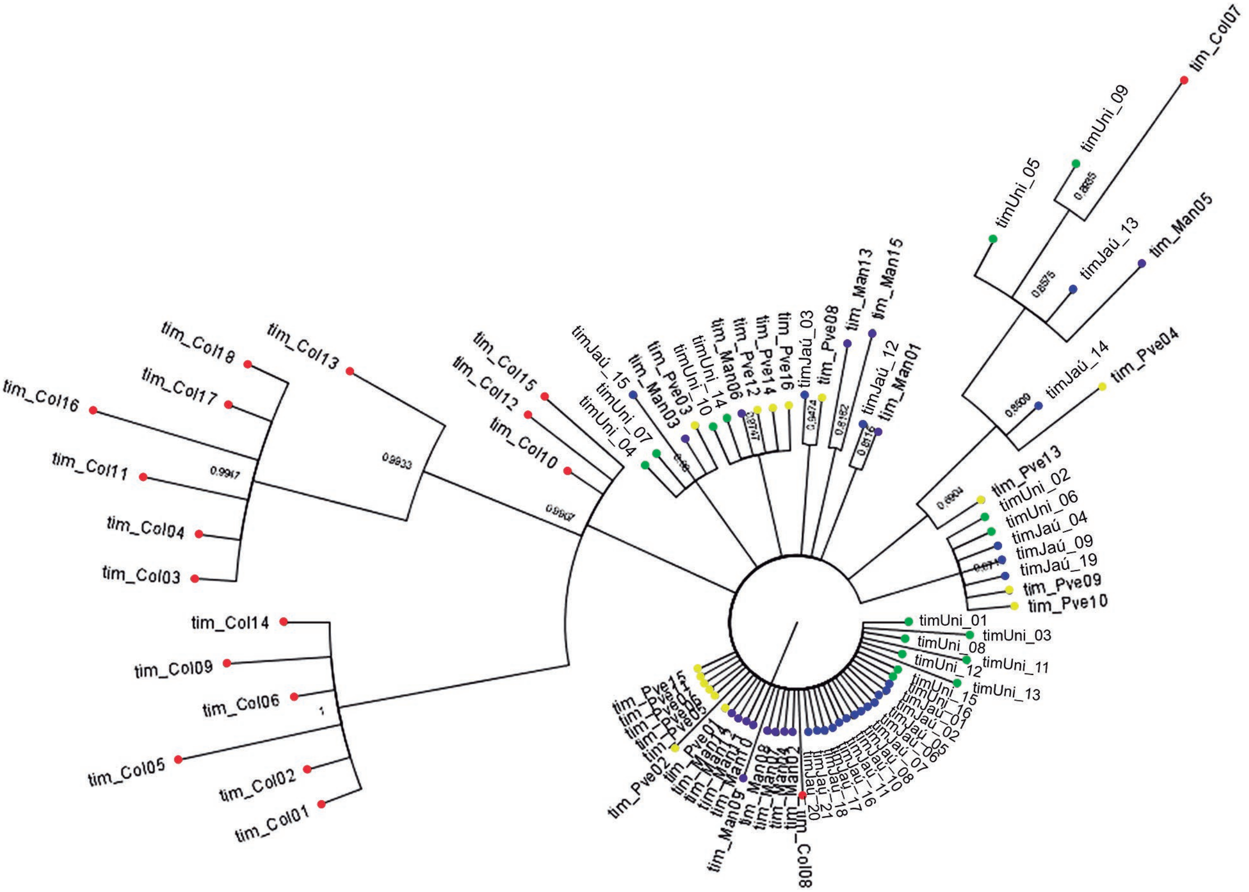

Fig. 10: Bayesian phylogenetic Kimura 2 parameters tree to *tim Anopheles darlingi* populations. In green: Unini River, blue: Jaú River, yellow: Porto Velho, purple: Manaus; red: Colombia. The numbers at the nodes represent the Bayesian posterior.

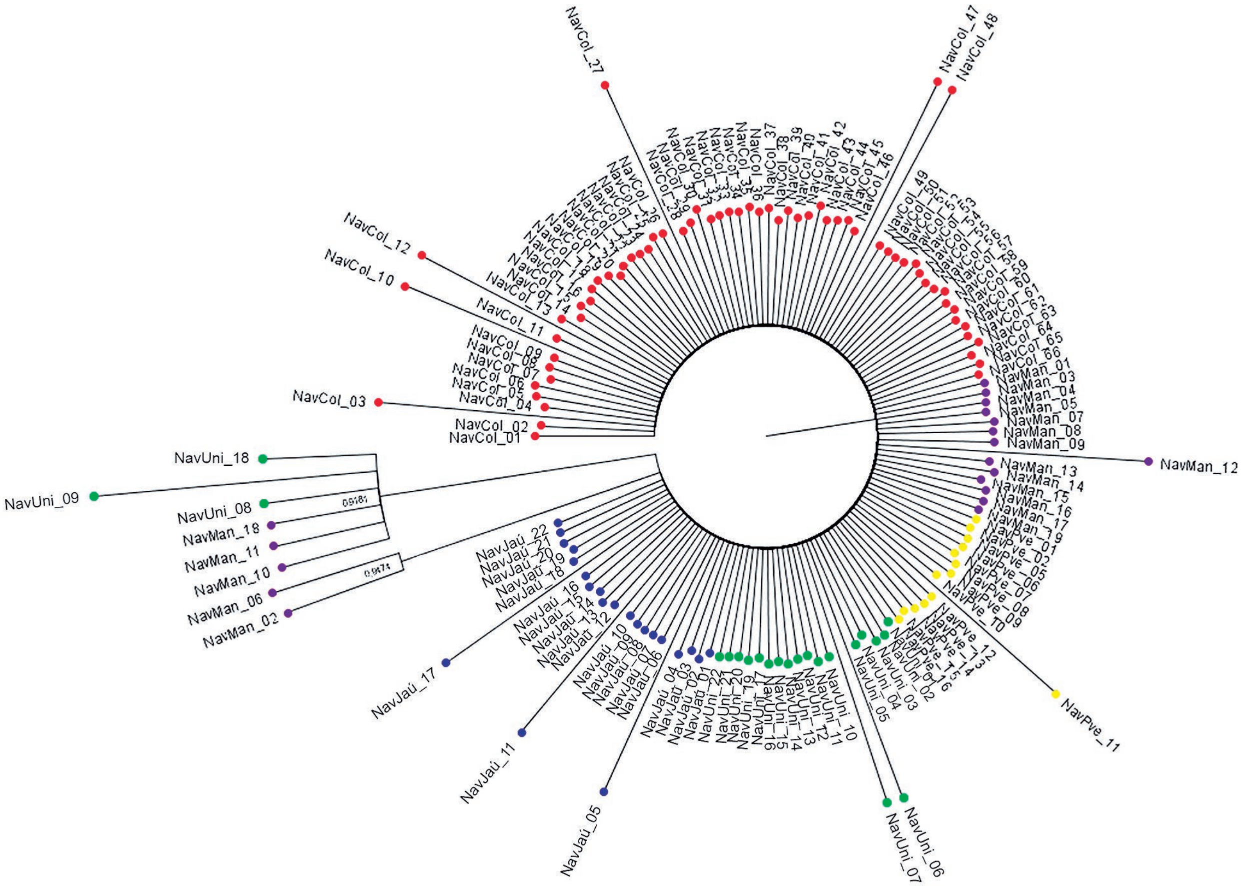

Fig. 11: Bayesian phylogenetic Hasegawa-Kishino-Yano tree to *Na$_V$ Anopheles darlingi* populations. In green: Unini River, blue: Jaú River, yellow: Porto Velho, purple: Manaus; red: Colombia. The numbers at the nodes represent the Bayesian posterior.

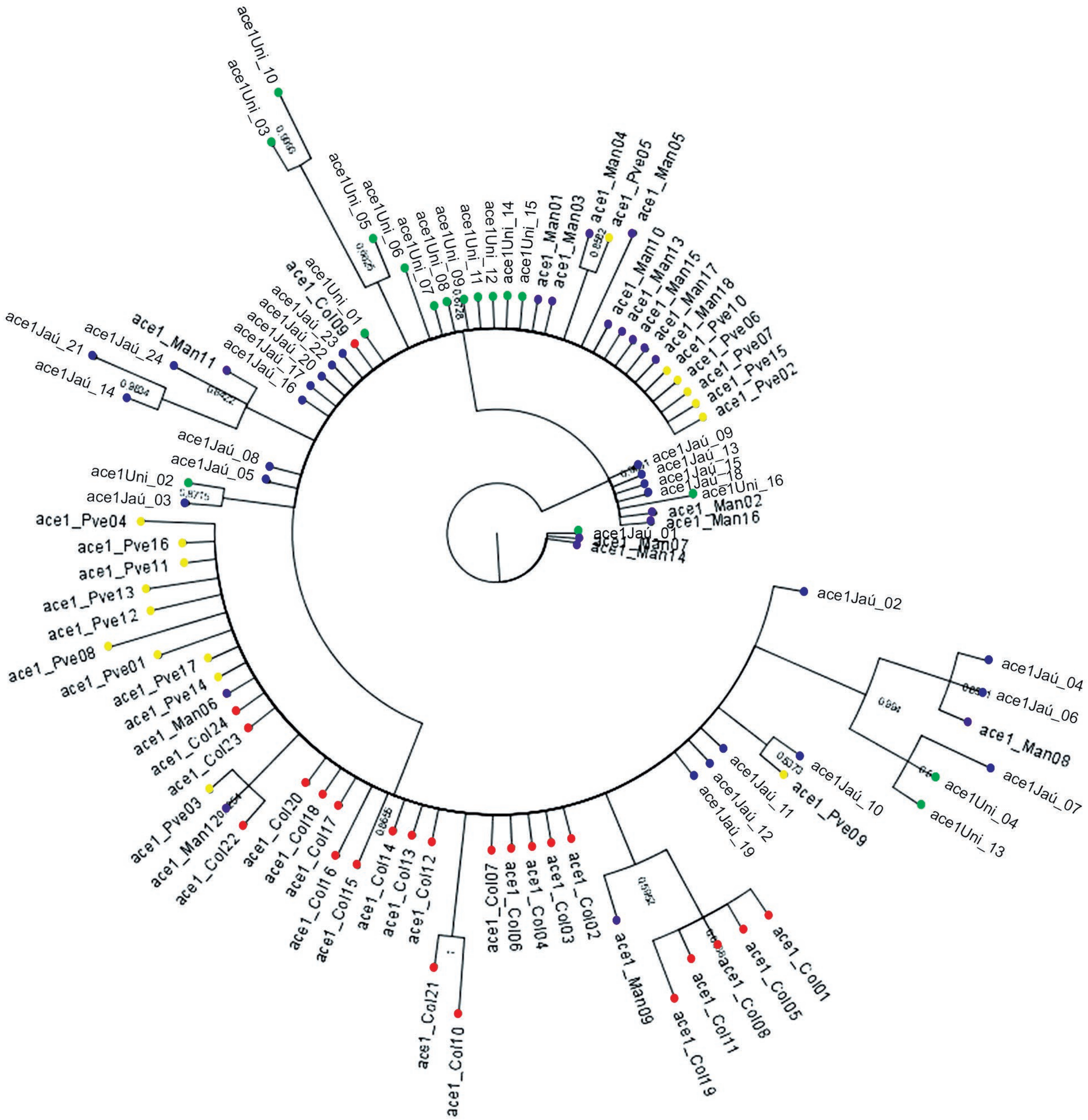

Fig. 12: Bayesian phylogenetic Tamura 3 parameters tree to *ace-1 Anopheles darlingi* populations. In green: Unini River, blue: Jaú River, yellow: Porto Velho, purple: Manaus; red: Colombia. The numbers at the nodes represent the Bayesian posterior.
